# A Non-Canonical Role for IRE1α Links ER and Mitochondria as Key Regulators of Astrocyte Dysfunction: Implications in Methamphetamine use and HIV-Associated Neurocognitive Disorders

**DOI:** 10.3389/fnins.2022.906651

**Published:** 2022-06-17

**Authors:** Jessica Proulx, Satomi Stacy, In-Woo Park, Kathleen Borgmann

**Affiliations:** Department of Microbiology, Immunology and Genetics at University of North Texas Health Science Center, Fort Worth, TX, United States

**Keywords:** mitochondria-associated ER membranes, unfolded protein response, metabolic function, neuroinflammation, astrogliosis, neurodegeneration

## Abstract

Astrocytes are one of the most numerous glial cells in the central nervous system (CNS) and provide essential support to neurons to ensure CNS health and function. During a neuropathological challenge, such as during human immunodeficiency virus (HIV)-1 infection or (METH)amphetamine exposure, astrocytes shift their neuroprotective functions and can become neurotoxic. Identifying cellular and molecular mechanisms underlying astrocyte dysfunction are of heightened importance to optimize the coupling between astrocytes and neurons and ensure neuronal fitness against CNS pathology, including HIV-1-associated neurocognitive disorders (HAND) and METH use disorder. Mitochondria are essential organelles for regulating metabolic, antioxidant, and inflammatory profiles. Moreover, endoplasmic reticulum (ER)-associated signaling pathways, such as calcium and the unfolded protein response (UPR), are important messengers for cellular fate and function, including inflammation and mitochondrial homeostasis. Increasing evidence supports that the three arms of the UPR are involved in the direct contact and communication between ER and mitochondria through mitochondria-associated ER membranes (MAMs). The current study investigated the effects of HIV-1 infection and chronic METH exposure on astrocyte ER and mitochondrial homeostasis and then examined the three UPR messengers as potential regulators of astrocyte mitochondrial dysfunction. Using primary human astrocytes infected with pseudotyped HIV-1 or exposed to low doses of METH for 7 days, astrocytes had increased mitochondrial oxygen consumption rate (OCR), cytosolic calcium flux and protein expression of UPR mediators. Notably, inositol-requiring protein 1α (IRE1α) was most prominently upregulated following both HIV-1 infection and chronic METH exposure. Moreover, pharmacological inhibition of the three UPR arms highlighted IRE1α as a key regulator of astrocyte metabolic function. To further explore the regulatory role of astrocyte IRE1α, astrocytes were transfected with an IRE1α overexpression vector followed by activation with the proinflammatory cytokine interleukin 1β. Overall, our findings confirm IRE1α modulates astrocyte mitochondrial respiration, glycolytic function, morphological activation, inflammation, and glutamate uptake, highlighting a novel potential target for regulating astrocyte dysfunction. Finally, these findings suggest both canonical and non-canonical UPR mechanisms of astrocyte IRE1α. Thus, additional studies are needed to determine how to best balance astrocyte IRE1α functions to both promote astrocyte neuroprotective properties while preventing neurotoxic properties during CNS pathologies.

## Introduction

According to the World Health Organization and the Joint United Nations Programme on human immunodeficiency virus (HIV)/AIDS, there are nearly 38 million individuals living with HIV/AIDS worldwide with an estimated 1.5 million newly infected individuals annually. Thanks to the development of the antiretroviral therapy (ART), HIV diagnosis is no longer a death sentence; however, this increases the global burden of people living with HIV (PLWH). Moreover, poor ART penetration to the central nervous system (CNS) allows persistent low levels of HIV replication and chronic neuroinflammation, which lead to the manifestation of HIV-associated neurocognitive disorders (HAND). These individuals are afflicted by different spectra of cognitive impairments and inference with daily functioning often referred to as “accelerated aging.” Even in the post ART era, approximately 30 – 70% of PLWH suffer from HAND depending on various combinational factors, including toxicity of ART itself, various sociodemographic disparities, comorbid health complications, and substance use disorders, which are disproportionately elevated among HIV-infected individuals (Parikh et al., 2012; Blackard and Sherman, 2021). As a common comorbidity among HIV-1 infected individuals (METH)amphetamine use can leave patients vulnerable to impaired immune function, insufficient adherence and resistance to treatment, and enhanced viral replication and infectivity (Ellis et al., 2003; Colfax et al., 2007; Liang et al., 2008; Toussi et al., 2009; Salamanca et al., 2014; Passaro et al., 2015; Basova et al., 2018; Skowronska et al., 2018). The use of METH can have long-lasting consequences on CNS homeostasis (Volkow et al., 2001; Hoefer et al., 2015; Jablonski et al., 2016). The combined neurological complications of HIV-1/METH comorbidity include increased excitotoxicity, oxidative damage, neuroinflammation, blood brain barrier (BBB) and neuronal injury, and neurocognitive impairment, which in turn impact the development and severity of HAND (Rippeth et al., 2004; Kesby et al., 2015; Soontornniyomkij et al., 2016; Fattakhov et al., 2021).

Astrocytes are one of the most abundant glial cells in the brain and are critical for CNS homeostasis. Paramount astrocyte functions include maintaining the integrity of BBB and participating in tripartite communication for proper neurotransmission. Moreover, astrocytes provide essential metabolic, antioxidant, and neurotrophic support to neurons to promote neuronal function and survival (Ricci et al., 2009; Chen et al., 2020). The consensus of the scientific community is that at least some human astrocytes are infected by HIV-1. An infection rate of even 1% would correlate to 0.4 – 1.3 billion HIV-1+ astrocytes in a human brain, which could have widespread consequences on neuronal survival, BBB permeability, and neuroinflammation. Astrocytes can not only serve as reservoirs for infection, but can also undergo activation, or “reactive astrogliosis” leading to phenotypic shifts in function (Edara et al., 2020a; Li et al., 2020). In the context of METH, astrocytes can become activated and remain reactive for extended periods of time even after withdrawal (Narita et al., 2008; Friend and Keefe, 2013; Bortell et al., 2017). The functional changes of astrogliosis are often associated with a more inflammatory and/or neurotoxic phenotype that can disrupt their ability to maintain CNS homeostasis and provide essential neuroprotective support to neurons. Indeed, reactive astrocytes are a central hallmark of many forms of neuropathology (Li et al., 2019; Matias et al., 2019). Identifying the underlying mechanisms that regulate astrocyte dysfunction during both HIV-1 infection and chronic METH exposure will illuminate therapeutic targets that can promote a disease preventative or neuroprotective phenotype during CNS pathology.

Cooperation between the endoplasmic reticulum (ER) and mitochondria is essential for the maintenance and restoration of cellular homeostasis. In fact, direct contact sites between these organelles termed mitochondria-associated ER membranes (MAMs) have been identified as critical intracellular hubs for determining cellular function and survival, especially during stress (Bravo et al., 2012; Filadi et al., 2017). Notably, the ER-mitochondria interface is a key regulator of mitochondrial physiology during both basal and stress-induced conditions (Bravo et al., 2012; Vannuvel et al., 2013; Rainbolt et al., 2014; Lebeau et al., 2018). For example, calcium ion transfer from the ER to mitochondria is vital for mitochondrial respiration and ATP synthesis (Bravo et al., 2012; Filadi et al., 2017). Commonly accepted MAM-associated calcium transporters include inositol 1,4,5-trisphosphate receptor (IP_3_R) on the ER membrane, voltage-dependent anion channel 1 (VDAC1) on the outer mitochondrial membrane, and mitochondrial calcium uniporter (MCU) on the inner mitochondrial membrane. However, recent studies focusing on the kinetics of astrocyte mitochondrial calcium influx confirmed a strong dependency on ER calcium stores but illuminated a minimal contribution of MCU (Huntington and Srinivasan, 2021). In addition, the three unfolded protein response (UPR) arms that are classically activated during ER stress demonstrate distinct contributions to both MAM regulation and mitochondrial homeostasis, beyond their classical UPR/ER stress functions. Briefly, protein kinase RNA-like endoplasmic reticulum kinase (PERK) is a key regulator of MAM formation as well as mitochondrial dynamics and bioenergetics (Verfaillie et al., 2012; Rainbolt et al., 2014; van Vliet and Agostinis, 2016; Lebeau et al., 2018; Balsa et al., 2019). Inositol-requiring protein 1α (IRE1α), commonly associated with cellular responses to infections or inflammation, is also implicated in ER-mitochondrial calcium transfer and regulation of mitochondrial respiration through association with IP_3_R on the ER membrane (Son et al., 2014; Carreras-Sureda et al., 2019) or sigma-1 receptor (σ-1R) in the ER lumen (Mori et al., 2013). Activating transcription factor 6 (ATF6) is known to both interact with and be regulated by the key MAM tethering protein vesicle-associated membrane protein-associated protein B (VAPB) (Gkogkas et al., 2008). Moreover, ATF6 regulates lipid biosynthesis and ER expansion, suggesting a possible interplay in MAM-mediated lipid homeostasis and ER-mitochondrial physiology (Bommiasamy et al., 2009).

The complete composition and function of MAMs remain unclear and can vary across cell types (Herrera-Cruz and Simmen, 2017; Janikiewicz et al., 2018; Moltedo et al., 2019). Modifications in MAM tethering and activity are implicated in a number of neurodegenerative diseases such as Alzheimer’s disease, Parkinson’s disease, and amyotrophic lateral sclerosis (Area-Gomez et al., 2012; Hedskog et al., 2013; Area-Gomez and Schon, 2017; Erpapazoglou et al., 2017; Rodriguez-Arribas et al., 2017; Leal et al., 2018) but have not yet been investigated in HAND or substance use disorders. Indeed, ER and oxidative stress, mitochondrial dysfunction, and calcium dysregulation are three MAM-associated disturbances that characterize neurodegenerative pathologies (Brown and Naidoo, 2012; Muller et al., 2018), including HAND, which our group recently reviewed (Proulx et al., 2021). The forefront of MAM research in the brain has largely been focused on neurons or whole brain tissues (Hedskog et al., 2013; Erpapazoglou et al., 2017; Rodriguez-Arribas et al., 2017; Leal et al., 2018). However, recent studies have demonstrated that astrocyte ER-mitochondrial contact and communication are critical regulators and potential therapeutic targets for astrocyte-mediated vascular remodeling (Gobel et al., 2020) and synaptic homeostasis (Serrat et al., 2021). Further exploration of astrocyte MAMs in health and disease will provide unique insights into astrocyte biology and inter-organelle communication to better understand how to regulate reactive astrocyte dysfunction. Moreover, METH- and HIV-1-relevant stimuli can induce ER stress and alter mitochondrial function, health, and/or morphology in astrocytes (Borgmann and Ghorpade, 2015, 2017; Fan and He, 2016; Shah and Kumar, 2016; Shah et al., 2016; Nooka and Ghorpade, 2017, 2018), suggesting an important role of the astrocyte ER-mitochondrial interface during HIV-1 infection and METH exposure. However, these findings have varied substantially across difference models.

Indeed, defects in mitochondrial bioenergetics, biogenesis, dynamics, degradation, integrity and transport are all hallmarks of HAND pathology (Haughey and Mattson, 2002; Huang et al., 2012; Avdoshina et al., 2016; Area-Gomez and Schon, 2017; Nooka and Ghorpade, 2017; Natarajaseenivasan et al., 2018; Ojeda et al., 2018; Rozzi et al., 2018; Teodorof-Diedrich and Spector, 2018; Thangaraj et al., 2018; Fields and Ellis, 2019; Khan et al., 2019; Rawat et al., 2019; Swinton et al., 2019). However, prior research into mitochondrial dysfunction in HAND has primarily concentrated on neurons or whole brain tissues, and results have varied substantially across different models (Avdoshina et al., 2016; Fields et al., 2016; Rozzi et al., 2017; Wang et al., 2017; Teodorof-Diedrich and Spector, 2018; Fields and Ellis, 2019). Specific investigations in astrocytes have suggested that HAND-relevant stimuli can induce mitochondrial depolarization and oxidative stress (Ton and Xiong, 2013; Nooka and Ghorpade, 2017, 2018; Natarajaseenivasan et al., 2018; Ojeda et al., 2018; Fields and Ellis, 2019). Among these findings, it was reported that astrocytes undergo a distinct metabolic shift in the presence of HIV-1 protein transactivator of transcription (Tat) alone and in combination with cocaine – a shift that impaired their capacity to provide essential metabolites to neurons and promoted neuroinflammation (Natarajaseenivasan et al., 2018). The use of several different HIV-1 models (including restrictive and active infection, exposure to infectious HIV-1 or HIV-1 proteins and proinflammatory cytokine treatment) reinforce these findings; however, the mechanisms or pathways mediating HIV-1-induced astrocyte dysfunction remain unknown.

In the context of METH exposure, it is well-known that METH can impair electron transport chain (ETC) function (Burrows et al., 2000a,b; Brown et al., 2005; Feier et al., 2012). In astrocytes, acute METH exposure with HIV-1 glycoprotein 120 exposure increased oxidative stress (Shah et al., 2013). Moreover, our lab has reported disproportionately augmented astrocyte mitochondrial oxygen consumption rate (OCR) compared to ATP levels following chronic METH exposure; in parallel, METH increased astrocyte antioxidant capacity and oxidative burden (Borgmann and Ghorpade, 2017). At least two METH receptors in astrocytes have been identified, trace amine associated receptor 1 and σ-1R (Cisneros and Ghorpade, 2012, 2014; Zhang et al., 2015), which could regulate some METH-associated dysregulation of astrocyte function. However, the METH experimental models have focused primarily on acute high doses.

Both UPR and calcium signaling have been recognized as potential perpetrators of astrocyte dysfunction during HAND pathology (Fan and He, 2016; Shah and Kumar, 2016; Nooka and Ghorpade, 2017, 2018; Natarajaseenivasan et al., 2018). In fact, inhibition of ER/UPR signaling is seen to reverse astrocyte-mediated neurotoxicity, apoptotic signaling, and mitochondrial dysfunction provoked by HIV-1 (Fan and He, 2016; Ma et al., 2016; Shah and Kumar, 2016). Moreover, manipulation of astrocyte intracellular calcium signaling provides resistance to HIV-1-induced ER stress and/or mitochondrial dysfunction (Nooka and Ghorpade, 2017; Natarajaseenivasan et al., 2018). Specifically, targeting MCU on the inner mitochondrial membrane to prevent mitochondrial calcium uptake when human astrocytes are challenged by HIV-1 Tat and/or cocaine restores neurotrophic mitochondrial function and replenishes astrocyte provision of essential metabolites to neurons (Natarajaseenivasan et al., 2018). However, as stated above, MCU may have a minimal contribution on astrocyte ER-mitochondrial calcium transfer, thus alternative targets need to be explored for therapeutic application (Huntington and Srinivasan, 2021). For example, suppression of astrocyte VDAC1 on the outer mitochondrial membrane was able to reverse HIV-1 Tat-induced release of ATP, subsequently rescuing neurons from astrocyte-mediated neurotoxicity (Fatima et al., 2017). Mortalin [*a.k.a.* glucose-regulated protein 75 kDa (grp75)] is a cytosolic scaffold protein between IP_3_R and VDAC1 to regulate ER-mitochondrial calcium transfer. Overexpression of mortalin/grp75 in astrocytes expressing HIV-1 Tat was able to prevent astrocyte mitochondrial dysfunction and fragmentation and protect neurons from astrocyte-mediated neurotoxicity by reducing the release of excess ATP, inflammatory cytokines, and extracellular glutamate (Priyanka et al., 2020). Altogether, these findings strongly support UPR signaling and/or ER-mitochondrial calcium transfer as potential regulators for astrocyte dysfunction and astrocyte-associated neuronal damage. Furthermore, a study in neurons exploring a mutated MAM tethering protein, mitofusion 2 (MFN2), reported a restoration of MAM tethering and mitochondrial dynamics, thereby providing resistance to neurite degeneration. Specific mechanisms involved in neuronal protection were preventing ER stress or activating σ-1R, a key regulator of the calcium transfer from the ER to the mitochondria (Bernard-Marissal et al., 2019).

While ER and oxidative stress are known outcomes of both METH and HIV-1 cytotoxicity, the connection between these outcomes has primarily been explored in the context of apoptosis. Interestingly, the resiliency of human astrocytes during METH- and HIV-1- relevant exposures supports the capacity of quality control mechanisms to enable survival under stressed conditions. However, as HAND and METH use disorders are chronic conditions, prolonged astrocyte activation may become neuropathic. Our investigations are specifically targeted toward elucidating novel signaling pathways linking ER and mitochondria during *chronic* METH exposure and HIV-1 *infection* in astrocytes. We then wanted to evaluate ER/MAM-associated molecules as potential regulators of astrocyte dysfunction during HIV-1/METH neuropathology. These findings demonstrate a dysregulated ER and mitochondrial homeostasis following HIV-1 infection and METH exposure in primary human astrocytes. We then further highlight UPR messenger, IRE1α, as a key signaling molecule for astrocyte mitochondrial respiration, glycolytic function, morphological activation, inflammation, and glutamate uptake. We propose further exploration into the therapeutic application of IRE1α as a central regulator of astrocyte dysfunction to help optimize the coupling between astrocytes and neurons and ensure neuronal fitness during a neuropathic challenge.

## Materials and Methods

### Primary Human Astrocyte Cultures

Primary human astrocytes were obtained from first or second trimester brain tissues from a biorepository at the University of Washington and in full compliance with local, federal, and National Institutes of Health (NIH) ethical guidelines. Written informed consent was obtained from all donors. Isolation and characterization of astrocyte cultures are previously described (Gardner et al., 2006; Borgmann and Ghorpade, 2017; You et al., 2020). Fresh and cryopreserved astrocyte cultures were used experimentally between passages two and seven. All experiments were replicated in three or more astrocyte cultures isolated from biologically distinct biospecimens.

### METH Treatment

Astrocytes were treated with METH (cat # M8750, Sigma-Aldrich, St. Louis, MO, United States) for acute calcium signaling (250 μM; 5 min), acute protein expression (5 μM; 8 h), or chronic assessments (50 – 250 nM; 7 days). Dose and time kinetics were determined based on the physiological peak (6 μM – 2 mM) and prolonged METH ranges (60 – 600 nM) found *in vivo* and our previous investigations (Rivière et al., 2000; Won et al., 2001; Cisneros and Ghorpade, 2012, 2014; Borgmann and Ghorpade, 2015, 2017; Shah and Kumar, 2016).

### Pseudotyped HIV-1

Human embryonic kidney (HEK) 293 T cells were obtained from the American Type Culture Collection (Manassas, VA, United States) and frozen at P4 according to their cryopreservation procedures. HEK 293 cells were plated in T75 flasks at 50% confluency and incubated at 37°C and 5% CO_2_ overnight. Cells were co-transfected with HIV-1 infectious molecular clone (pNL4-3; cat # ARP-114, NIH HIV Reagent Program, Manassas, VA, United States, contributed by Dr. M. Martin) and p-human elongation-factor (pHEF)-vesicular stomatitis virus glycoprotein (VSVg; plasmid # 22501, Addgene, Watertown, MA, United States, a gift from Dr. Sergey Kasparov) by calcium phosphate precipitation per CalPhos Mammalian Transfection kit instructions (Clontech Laboratories, Inc., Mountain View, CA, United States) (Canki et al., 2001; Ojeda et al., 2018; Edara et al., 2020a). A total of 5 μg of plasmid DNA (pDNA) at a ratio of 1:1.5 (pHIV-1:VSVg) was added dropwise to each flask in a 2 mL precipitate of solution A (2 M calcium solution + pDNA diluted in sterile water) and solution B (2x HEPES solution, 1:1). Cells were incubated overnight, washed three times with PBS, and fresh culture media was added. Supernatants were collected 48 h post-wash, and pseudotyped HIV-1 was quantified by reverse transcriptase (RT) activity *via* radiometric RT assay (Edara et al., 2020a). Pseudotyped HIV-1 doses ranging from 100 to 1,000 units RT were tested. The optimal dose of pseudotyped HIV-1 for the present study (500 RT) was selected based on confirmed integration, detectable levels of viral protein expression, and minimal cytotoxicity to best mimic chronic *in vivo* HIV-1 astrocyte infection.

### HIV-1 DNA Integration Assay

Astrocytes were plated in six-well plates at 1 million cells per well and treated with or without pseudotyped HIV-1 (500 RT). Cells were incubated overnight, washed three times with PBS, and fresh media was added. Astrocytes were collected using 0.05% Trypsin-EDTA (cat # T3924; Sigma-Aldrich) 7 days post infection and washed with PBS prior to DNA isolation using QIAamp DNA Micro Kit per manufacturer’s instructions (Qiagen, Germany). Successful integration of HIV-1 was determined by a nested, two-step PCR integration assay as previously described (Edara et al., 2020a). HIV-1 lymphadenopathy-associated virus-infected 8E5 cells (8E5, cat # ARP-95, NIH HIV Reagent Program, contributed by Dr. Thomas Folks) were used as a positive control. Briefly, DNA was amplified with sequence-specific primers for Alu (5′-TCC CAG CTA CTC GGG AGG CTG AGG-3′) – gag (5′-CCT GCG TCG AGA GAG CTC CTC TGG-3′) using Phusion high-fidelity PCR kit (cat # F553S; Thermo Fisher Scientific, Waltham, MA, United States). The first PCR product was diluted 10-fold and measured in a second PCR specific for R/U5 DNA with sense primer M667 (5′-GGC TAA CTA GGG AAC CCA CTG C-3′) and antisense primer AA55 (5′-CTG CTA GAG ATT TTC CAC ACT GAC-3′). The final PCR product was visualized with a FluorochemQ gel imaging station (ProteinSimple, San Jose, CA, United States) following electrophoresis on a 1% agarose gel with ethidium bromide.

### Treatment With Pharmacological Inhibitors

Astrocytes were treated with inhibitors [ATF6 (AEBSF; cat # A8456, Sigma-Aldrich; 100 μM), PERK (GSK2606414; cat # 516535, MilliporeSigma, Burlington, MA, United States; 1 μM), IRE1α (STF-083010; cat # 412510, MilliporeSigma, 60 μM; 4 μ8c; cat # 50-136-4583, Thermo Fisher Scientific, 60 μM)] 3 h prior to Seahorse assessment. Dose and time kinetics were selected for the inhibitors based on previous studies (Bravo et al., 2011; Liu et al., 2013, 2018; Cisneros and Ghorpade, 2014; Jiang et al., 2017; Nooka and Ghorpade, 2017).

### Transfection

Transfections were performed using the Amaxa P3 primary cell 96-well kit, nucleofector and shuttle attachment (cat # V4SP-3960, Lonza, Walkersville, MD, United States) per manufacturer’s instructions with modification as previously published (Nooka and Ghorpade, 2017). Briefly, 250 – 500 ng of plasmid DNA was transfected per 1.6 million astrocytes in 20 μL of nucleofection reagent. A circularly permutated green fluorescent protein (GFP) with a calmodulin tag was constructed as an ultrasensitive calcium sensor (GCaMP6s) and gifted from Dr. Douglas Kim (pGP-CMV-GCaMP6s, plasmid # 40753, Addgene) (Chen et al., 2013). The same protocol was used for IRE1α overexpression (cat # SC309043, OriGene, Rockville, MD, United States) and the backbone plasmid (cat # PCMV6XL5, OriGene). Astrocytes were plated and allowed to recover for 24 – 48 h before treatment or downstream assessments.

### Interleukin 1β Treatment

After recovery from IRE1α overexpression or backbone transfection, astrocytes were then treated with interleukin 1β (IL-1β) (20 ng/mL; 24 h; cat # 201-LB, R&D Systems, Minneapolis, MN, United States) for functional analyses based on a decade-long protocol that has been optimized for astrocyte characterization (You et al., 2020).

### Calcium Signaling

Following transfection with GCaMP6s, astrocytes were plated at 100,000 cells per well in tissue culture treated, six channel μ-slides (0.4 VI, cat # 80606, ibidi, Madison, WI, United States). Each condition was performed in duplicate wells for each biological donor with a minimum of 20 cells imaged per donor. Changes in calcium flux were imaged with a 20x objective at excitation of 450 – 490 nm and emission of 593 – 668 nm, using time series confocal microscopy *via* Carl Zeiss LSM 510 (Jena, Germany) (Nooka and Ghorpade, 2017). Time-lapse micrographs were acquired every 500 ms for 5 min. Astrocytes were treated with control media or METH (250 μg/ml) at 10 s (20 cycles) then ionomycin (10 μM) at 225 s (450 cycles). Analysis was performed using Fiji ImageJ software (Version: 2.0.0-rc-69/1.52i; National Institutes of Health, Bethesda, MD, United States). Individual cells were outlined, and the change in fluorescence was calculated by: ΔF = (F-F_0_)/(F_*max*_-F_0_), where F is the fluorescence intensity at any given time; F_0_ is the baseline (1 – 20 cycles) fluorescence intensity, and F_*max*_ is the maximum fluorescence intensity when exposed to ionomycin (450 – 600 cycles). Calcium flux line tracings illustrate the ΔF at any given time point. Area under the curve (AUC) was calculated by the sum of ΔF between METH/media and ionomycin treatment (cycles 20 – 450).

### Protein Expression *via* Simple Wes

Astrocytes were plated 48 h prior to collection in six-well plates at 2 million cells per well. Lysates were collected using mammalian protein extraction buffer (MPER, cat # PI78505, Thermo Fisher Scientific) with protease and phosphatase inhibitors (cat # P8340, cat # P0044 and cat # P2850, Sigma-Aldrich). Protein concentrations were quantified *via* bicinchoninic acid (BCA) assay per manufacturer’s instructions (cat # 23225, Thermo Fisher Scientific). Lysates were then diluted in 0.1X Wes Sample Buffer to 2 μg/μL for HIV-1 protein assessment or 0.5 μg/μL for all other targets. Protein expression was determined using Simple Wes in 12 – 230 kDa Separation Modules (cat # SM-W004, ProteinSimple) per manufacturer’s instructions. Briefly, sample buffer, loading dye, ladder, primary antibody dilution buffer, secondary antibodies, and all other necessary reagents for separation module set-up were provided by manufacturer with instructions for reconstitution and use. Primary antibody dilutions were standardized for each target. An automated capillary system performs all sample separation, wash, and immunolabeling steps to provide quantitative, size-based data based on changes in chemiluminescence. Compass for SW software (Version 4.0.0) was used to collect digitalized data and blot images. Targets included: HIV-1 negative factor protein (Nef; cat # ARP-3689, HIV Reagent Program, contributed by Dr. James Hoxie; 1:25, ∼30 kDa), HIV-1 capsid protein (p24; cat # ab43037, Abcam, Cambridge, United Kingdom; 1:25, ∼32 kDa), binding immunoglobulin protein (BiP; clone C50B12, cat # 3177, Cell Signaling Technology, Danvers, MA, United States; 1:250, ∼71 kDa), ATF6 (clone D4Z8V, cat # 65880, Cell Signaling Technology; 1:50, ∼117 kDa), PERK (clone D11A8, cat # 5683, Cell Signaling Technology; 1:50, 170 kDa), IRE1α (clone 14C10, cat # 3294, Cell Signaling Technology; 1:50, ∼130 kDa), and vinculin (clone E1E9V, cat # 13901, Cell Signaling Technology; 1:20,000, ∼117 kDa). Note: molecular weights can shift in Simple Wes compared to classical western blot.

### Mitochondria Bioenergetics

Metabolic profiles were performed using Seahorse XFe96 analyzer technology per Seahorse XF Cell Mito Stress Test Kit User Guide instructions and as previously described (cat # 103015-100, Agilent Technologies, Santa Clara, CA, United States) (Prah et al., 2019; Chaphalkar et al., 2020). Briefly, astrocytes were plated 48 h prior to Mito Stress Test in Seahorse 96-well plates at 25,000 – 30,000 cells per well. There was a minimum of 6 wells per condition for each biological donor. On the day of experiment, astrocyte media was exchanged with assay media 1 h before testing. During assay, consecutive injections of oligomycin (Oligo; 2 μM), carbonyl cyanide-4 (trifluoromethoxy) phenylhydrazone (FCCP; 2 μM), and rotenone/antimycin A (Rot/AA; 0.5 μM) were used to modulate components of the ETC allowing assessment of key parameters of mitochondrial function. Data were collected using Wave V2.6.1.56 software and exported with Seahorse XF Cell Mito Stress Test Report Generator.

### Cytotoxicity

Toxicity of the three UPR inhibitors was measured *via* extracellular lactate dehydrogenase (LDH) assay using Cytotoxicity Detection Kit (cat # 11644793001, Sigma-Aldrich). Astrocytes were plated in 48-well plates at 150,000 cells per well overnight. The next day, astrocytes were treated in triplicates with the UPR inhibitors for 3 h to model Seahorse timepoints. Duplicate supernatant collections (50 μL) per well (six total samples per experiment) were incubated 1:1 with LDH reaction buffer for 25 min in the dark per manufactures instructions. Absorbance was read at 490 nm with background correction at 650 nm.

### Immunocytochemical Staining

Astrocytes were plated in 48-well plates at 100,000 cells per well for 48 h prior to fixation with 1:1 acetone: methanol (24 h post IL-1β treatment). Fixed cells were then blocked and immunolabeled in 1 × PBS containing 2% BSA and 0.1% Triton X-100 (PBS, cat # BP3994; BSA, cat # BP1600-1, Thermo Fisher Scientific; Triton X-100, cat # X-100, Sigma-Aldrich). Primary antibodies specific to glial fibrillary acidic protein (GFAP; chicken, cat # 829401, BioLegend, San Diego, CA, United States, 1:700) and HIV-1 structural protein p24 (mouse, cat # ab9044, Abcam, 1:100) or IRE1α (clone 14C10, rabbit, cat # 3294, Cell Signaling Technology, 1:100) were incubated overnight at 4°C. Alexa Fluor secondary antibodies (488 nm, chicken, cat # A11042; 594 nm, mouse, cat # A11032; 594 nm, rabbit, cat # A11037; 1:400; Thermo Fisher Scientific) were incubated for 2 h at room temperature. Cells were washed between incubation steps. Nuclei were labeled with 4’,6-diamidino-2-phenylindole (DAPI; cat # D1306, 1:1000, 3 min, Thermo Fisher Scientific) prior to mounting and imaging. Fluorescent microscopy images were obtained on ECLIPSE Ti-4 using the NIS-Elements BR. 3.2 software (Nikon, Minato, Tokyo, Japan) to visualize the proteins and cellular morphology.

### Morphology Activation

Glial fibrillary acidic protein fluorescence intensity was measured using SpectraMax M5 plate reader (Molecular Devices, San Jose, CA, United States) following immunocytochemical staining. Excitation was set at 488 nm, emissions at 525 nm and auto fluorescence cutoff at 525 nm. DAPI fluorescence intensity was used to normalize GFAP per cell. Duplicate full well scans were quantified for each condition per biological donor. Astrocyte process length was measured using Fiji ImageJ software. Astrocytes were selected based on distinction of processes. For percent (%) morphological activation, astrocytes with constricted bodies and distinct processes were divided by the total number of astrocytes per image. Process length and percent morphological activation were quantified across three images per condition for each biological donor.

### Chemokine Expression

Astrocytes were plated in triplicates in 48-well plates at 150,000 cells per well. Supernatants were collected 48 h post plating (24 h post IL-1β treatment). Colorimetric enzyme-linked immunosorbent assays (ELISA) were performed according to the manufacturer’s instructions to quantify C-C motif chemokine ligand 2 (CCL2) and C-X-C motif chemokine ligand 8 (CXCL8) secretion levels in culture supernatants (CCL2, cat # DCP00; CXCL8, cat # D8000C, R&D Systems). Cells metabolize 3-(4,5-dimethylthiazol-2-yl)-2,5-diphenyltetrazolium bromide (MTT) into formazan crystals, which was used to assess metabolic activity as an indicator for cell number. MTT activity was used to normalize ELISA results (Edara et al., 2020a; You et al., 2020).

### Glutamate Clearance

Glutamate clearance assays in primary human astrocytes were performed as previously described and published as a bio-protocol (Edara et al., 2020a; You et al., 2020). Briefly, astrocytes were plated in triplicates in 48-well plates at 150,000 cells per well. After 24 h, astrocytes were treated with or without IL-1β (20 ng/mL). After an additional 24 h, media was exchanged (collected for ELISA) and glutamate (400 μM; cat # G8415, Sigma-Aldrich) in phenol red-free astrocyte medium was added to cultures. Wells without astrocytes were used as controls so that uptake/clearance could be assessed overtime by quantifying remaining glutamate per manufacturer’s instructions (Amplex Red glutamic acid/glutamate oxidase assay kit; Thermo Fisher Scientific). Supernatants for glutamate clearance were assessed 24 h post glutamate treatment (72 h post plating).

### Data Analysis and Interpretation

All experiments were performed in at least three separate biological donors. Data in graphs were analyzed and presented using GraphPad Prism (version 8.1.1, GraphPad Software, San Diego, CA, United States) as mean ± standard error of the mean with replicates from each donor compiled and represented as a single dot on graphs. For protein expression analysis following chronic conditions, each target was individually graphed and analyzed using paired *t*-tests. All other statistics were determined *via* one-way ANOVA followed by Fisher’s LSD test (calcium signaling) or Tukey’s post-test for multiple comparisons with *p* ≤ 0.05. Note: Fisher’s LSD test was used to account for ultra-sensitivity and variability of sensor across different biological donors.

## Results

**FIGURE 1:**
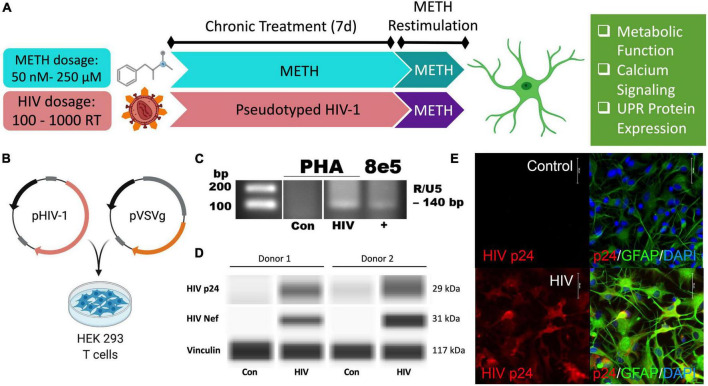
Pseudotyped HIV-1 infection in astrocytes is characterized by HIV-1 DNA integration and productive protein expression. **(A)** Schematic of the proposed experimental design to assess the effects of METH exposure and HIV-1 infection on astrocyte ER-mitochondrial homeostasis. **(B)** Pseudotyped HIV-1 was constructed by co-transfecting an HIV-1 plasmid (pHIV-1) with vesicular stomatitis virus glycoprotein plasmid (pVSVg) in HEK 293 T cells. **(C–E)** Primary human astrocyte (PHA) cultures were infected with **(C,D)** 500 RT pseudotyped HIV-1 for 5 days or **(E)** 1000 RT pseudotyped HIV-1 for 7 days followed by **(C)** HIV-1 DNA integration assay or **(D)** Simple Wes to detect expression of HIV-1 proteins p24 or Nef. Vinculin was used as an internal control. **(E)** Immunocytochemistry staining of HIV-1 protein p24 (red), astrocyte marker glial fibrillary acidic protein (GFAP, green) with nuclear DNA labeled with DAPI (blue). Experimental illustrations were made with BioRender.com.

As HAND is a chronic condition, changes in astrocyte phenotype were assessed 7 days following low dose METH exposure (50 or 250 nM) or post-infection to model the lingering low levels of METH in the CNS between binges of a regular user. Restimulation with acute high doses of METH (5 or 250 μM) was used to model the physiological peak of METH in the CNS during a binge to evaluate how chronic stress following METH exposure or HIV-1 infection shifts astrocyte responses to new stimuli. Subsequent phenotype studies evaluated changes in metabolic function, calcium signaling, and UPR protein expression (Figure 1A).

Astrocytes do not express the key receptor (CD4) required for conventional HIV-1 entry. However, astrocytes can undergo other means of HIV-1 infection such as direct cell-cell transfer of the virus *via* infected CD4+ T cells (Luo and He, 2015; Lutgen et al., 2020), whose trafficking into the CNS has been established (Mathe et al., 1997; Spudich et al., 2019). To investigate specifically how HIV-1 infection alters astrocyte phenotype, a pseudotyped HIV-1 was constructed by co-transfecting an HIV-1 plasmid (pHIV-1) with pVSVg in HEK 293 cells (Figure 1B). In VSV particles, VSVg is the predominant coat responsible for virus entry *via* membrane fusion. Thus, progeny pseudotyped HIV-1 virions will incorporate VSVg in their viral coat and permit entry independent of CD4 expression as previously described (Canki et al., 2001; Ojeda et al., 2018; Edara et al., 2020a). Notably, a T-tropic pHIV-1 strain (NL4-3) was used to model astrocytes infected *in vivo* by T-cell-mediated HIV-1 transfer.

HIV-1 infection of primary human astrocytes (PHA) was confirmed 5 days post-infection using a nested HIV-1 DNA integration assay (Figure 1C). As a positive control for integration, 8E5 cells were used as they are a T cell generated subclone that contains a single integrated copy of proviral DNA per cell (Desire et al., 2001; Wilburn et al., 2016). Expression of the HIV-1 structural protein p24 and the regulatory protein Nef was evident only in HIV-1 treated astrocytes and was illustrated in two astrocyte donors by Simple Wes (Figure 1D). Vinculin was used as a protein loading control. Expression of p24 (red) was visualized using immunocytochemistry in astrocytes stained with astrocyte marker, GFAP (green) following 7 days infection with pseudotyped HIV-1 (1000 RT) (Figure 1E). Notably, astrocyte staining also revealed morphological activation following infection as evident by more distinctive processes and cell body constriction. Moreover, pseudotyped HIV-1-infected astrocyte cultures had an elevated intensity in GFAP, which is a known indicator of astrogliosis and associates with increased neuropathology (Eng and Ghirnikar, 1994; Bettcher et al., 2021).

**FIGURE 2:**
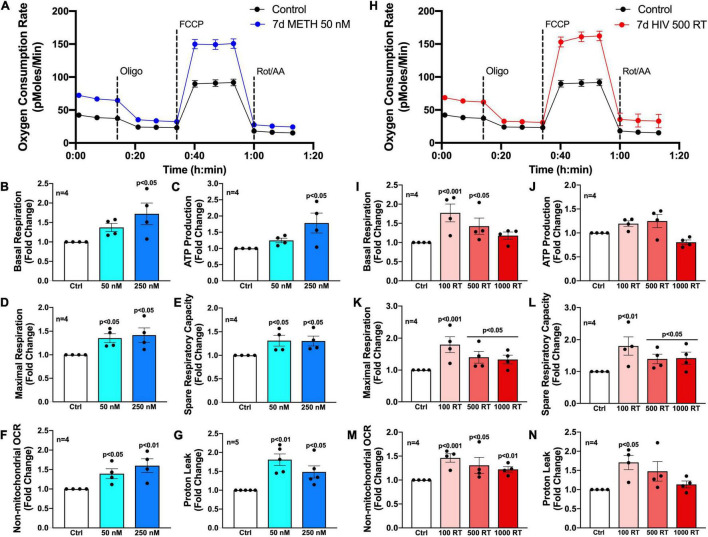
Chronic METH exposure and HIV-1 infection increase astrocyte metabolic activity. Astrocytes were treated with **(A–G)** METH (50 or 250 nM) or **(H–N)** infected with pseudotyped HIV-1 (100–1000 RT) for 7 days prior to Seahorse XF Cell Mito Stress Test. **(A,H)** Representative metabolic profile tracings from a single astrocyte donor are graphed over time. Fold changes in oxygen consumption rates (OCR) quantifying **(B,I)** basal respiration, **(C,J)** ATP production, **(D,K)** maximal respiration, **(E,L)** spare respiratory capacity, **(F,M)** non-mitochondrial OCR, and **(G,N)** proton leak were graphed for statistical comparisons. Individual dots on graphs represent the averaged data from a minimum of six replicates per biological donor. Statistical significance was determined by one-way ANOVA followed by Tukey’s *post hoc* for multiple comparisons.

To evaluate how chronic METH exposure or HIV-1 infection alters astrocyte metabolic phenotype, astrocytes were treated with METH (50 or 250 nM) (blue bars) or infected with pseudotyped HIV-1 (100–1000 RT) (red bars) for 7 days prior to assessing mitochondrial function *via* Seahorse XF Cell Mito Stress Test (Figure 2). Agilent Seahorse XF Analyzers measure real-time OCR and extracellular acidification rate (ECAR) to characterize mitochondrial respiration and glycolysis, respectively. The Cell Mito Stress Test is widely regarded as a “gold standard” for quantifying key parameters of mitochondrial function in cells. Using an injection sequence of three ETC modulators, the assay determines basal respiration, ATP-linked respiration, maximal respiration, spare respiratory capacity, non-mitochondrial respiration, and proton leak from calculated OCR readouts.

Primary human astrocytes exposed to METH (50 nM) for 7 days had elevated OCR overall compared to untreated controls as illustrated in an OCR line tracing from a representative astrocyte donor graphed over time (Figure 2A). Moreover, analysis of subsequent mitochondrial parameters demonstrated dose-dependent consequences following chronic METH (50 or 250 nM) exposure (Figures 2B–G). The basal energetic demand between conditions is reflected by baseline readouts prior to stress test modulator injections. The first injection of oligomycin (Oligo) inhibits ATP synthase (complex V) allowing quantification of ATP-linked respiration. While elevated, both basal respiration (Figure 2B) and ATP production (Figure 2C) were not statistically significant for the lower dose of METH at 50 nM but had approximately 1.75-fold increases at the higher METH dose at 250 nM (*p* < 0.05). The second injection of FCCP is an uncoupler allowing uninhibited (maximal) electron transport and respiratory capacity. The difference between basal and maximal respiration is used to calculate spare respiratory capacity and is an indicator of “cell fitness or flexibility.” Both maximal respiration (Figure 2D) and spare respiratory capacity (Figure 2E) were significantly elevated by chronic METH (50 or 250 nM) exposure (*p* < 0.05). Finally, the last injection is a combination of rotenone and antimycin A (Rot/AA), which inhibits complexes I and III, respectively and effectively shuts down ETC function allowing assessment of non-mitochondrial respiration. Non-mitochondrial respiration provides insight to oxygen-consuming enzymes outside that of the ETC, which may be contributing to metabolic demand (glycolysis, fatty acid oxidation, *etc*.). The difference between non-mitochondrial respiration and respiration remaining after ATP synthase inhibition indicates the proportion of proton leak between conditions and can be an indicator of mitochondrial damage. Like mitochondrial respiration, non-mitochondrial respiration (Figure 2F) was dose-dependently increased following chronic METH (50 or 250 nM) exposure (*p* < 0.05). Interestingly, proton leak (Figure 2G) was higher at the lower dose of METH (50 nM; 1.75-fold increase compared to control; *p* < 0.01) compared to 250 nM (1.5-fold increase compared to control; *p* < 0.05).

Like chronic METH exposure, HIV-1-infected astrocytes had an elevated OCR overall compared to untreated controls as illustrated in an OCR line tracing from a representative astrocyte donor graphed over time (Figure 2H). However, there was a reverse dose trend evident in HIV-1-infected astrocytes wherein the lower concentration of HIV-1 (100 RT) had a consistently higher increased OCR in key metabolic parameters compared to the higher HIV-1 doses (500 or 1000 RT) (Figures 2I–N). Indeed, 100 RT of pseudotyped HIV-1 induced 1.75-fold increases in basal respiration (Figure 2I; *p* < 0.001), maximal respiration (Figure 2K; *p* < 0.001), and spare respiratory capacity (Figure 2L; *p* < 0.01), while 500 RT doses induced 1.5-fold increases in the same parameters (*p* < 0.05). The highest HIV-1 dose (1000 RT) had similar effects as 500 RT on maximal respiration and spare respiratory capacity (*p* < 0.05) but did not significantly elevate basal respiration. All HIV-1 doses also significantly increased non-mitochondrial respiration (Figure 2M; ∼1.25–1.5-fold; *p* < 0.05), although less robustly than the effects on mitochondrial respiration. Notably, increased metabolic activity was not accompanied with changes in ATP production (Figure 2J) following infection at any dose tested suggesting increased respiratory demand without the energetic reward. It should also be noted that proton leak can be used as a mechanism to regulate the mitochondrial ATP production. While proton leak (Figure 2N) followed similar increased overall trends following HIV-1 infection, variable effects across separate biological donors translated to only the low HIV-1 dose (100 RT) to be statistically significant (*p* < 0.05).

Altogether, these findings demonstrate that low levels of METH or HIV-1 infection increase astrocyte metabolic activity. Increased basal respiration (Figures 2B,I), maximal respiration (Figures 2D,K) and spare respiratory capacity (Figures 2E,L) suggest that astrocyte mitochondria may need to overcompensate during conditions of chronic stress. Increased proton leak (Figures 2G,N) and lack of significant increases in ATP production (Figures 2C,J) suggest impaired mitochondrial integrity. Meanwhile, increased non-mitochondrial oxygen consumption (Figures 2F,M) supports a potential astrocyte metabolic shift. Regardless, the cause, purpose and outcome of these increased metabolic states are not fully understood.

**FIGURE 3:**
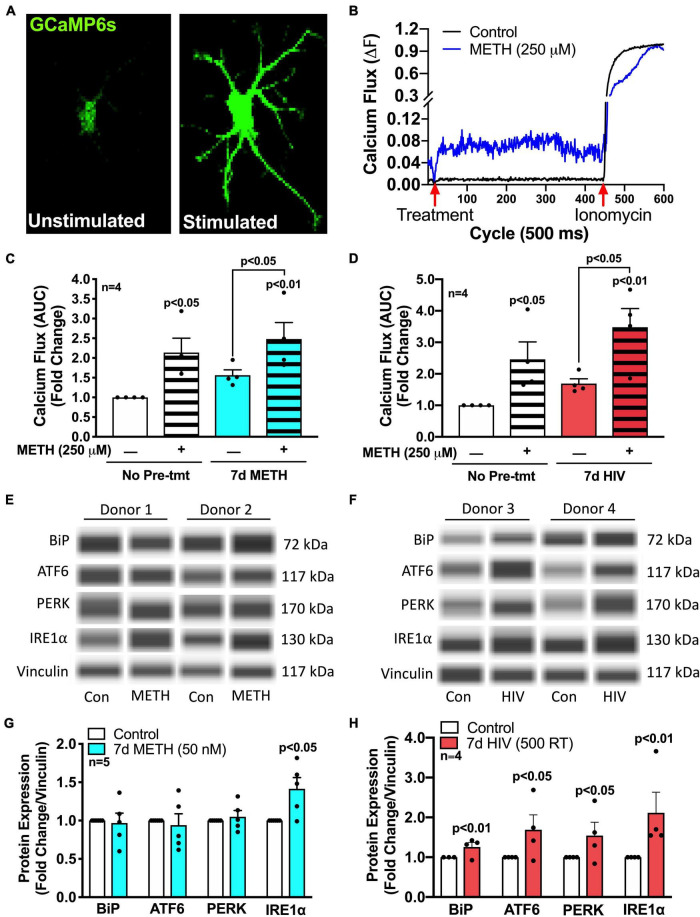
METH and HIV-1 upregulate ER/UPR signaling mediators. Astrocytes were treated with **(C,E)** METH (50 nM) for 7 days (blue bars) or **(D,F)** pseudotyped HIV-1 (500 RT) for 7 days (red bars) before **(A–D)** calcium imaging or **(E,F)** protein analysis *via* Simple Wes. **(A–D)** Astrocytes were transfected with a GFP-calmodulin calcium sensor (GCaMP6s) for 48 h prior to calcium flux analysis. **(A,B)** Time series confocal imaging was used to measure changes in fluorescence every 500 ms for a total of 5 min (600 cycles). **(B)** Representative calcium flux line tracings illustrate the change in astrocyte calcium flux (ΔF) at any given time point, with control media or METH (250 μM) at 20 cycles (10 s) and ionomycin (10 μM) at 450 cycles (225 s). Calcium flux was calculated by: ΔF = (F-F_0_)/(F_*max*_-F_0_), where F is the fluorescence intensity at any given time; F_0_ is the baseline (1 – 20 cycles) fluorescence intensity, and F_*max*_ is the maximum fluorescence intensity when exposed to ionomycin (450 – 600 cycles). **(C,D)** Area under the curve (AUC) was calculated by the sum of ΔF following treatment with control media or METH (250 μM) at 20 cycles (10 sec) and before ionomycin (10 μM) at 450 cycles (225 s). Individual dots represent the average AUC from a minimum of 20 cells per biological donor and are graphed as fold changes. One-way ANOVA was performed for statistical analysis followed by Fisher’s LSD test for stand-alone comparisons to account for sensitivity of calcium flux variation across different biological donors. **(E–H)** Protein expression of BiP, ATF6, PERK, and IRE1α was measured *via* Simple Wes post-treatment of **(E,G)** chronic (7 days) METH (50 nM) or **(F,H)** HIV-1 infection (7 days; 500 RT). **(E,F)** Representative blot images are illustrated from two separate biological donors per post-treatment paradigm. **(G,H)** Data from a minimum of four donors are compiled for graphical representation. Individual dots on graphs represent fold changes to vinculin for separate biological donors. Statistics were performed using ratio-paired *t*-tests for individual targets per condition.

Mitochondrial bioenergetics is regulated by the transfer of calcium from ER, which is the primary calcium source for astrocyte mitochondrial calcium fluxes (Huntington and Srinivasan, 2021). To measure changes in intracellular calcium flux following METH exposure or HIV-1 infection, astrocytes were transfected with a genetically modified GFP cytosolic calcium sensor (GCaMP6s) (Figures 3A–D; Chen et al., 2013; Nooka and Ghorpade, 2017). Time series confocal microscopy allowed fluorescent visualization of a single cell’s calcium flux post treatment (Figure 3A), and changes in fluorescence were graphed over time (Figure 3B and Supplementary Figure 1). To demonstrate variability across biological donors and conditions, ten individual cellular responses were graphed per condition in at least two separate donors (Supplementary Figure 1). The AUC, following stimulation with either media or METH, was then graphed for statistical comparisons (Figures 3C,D). In astrocytes, acute METH (250 μM, striped bars) increased astrocyte intracellular calcium flux by twofold versus untreated controls (*p* < 0.05). Interestingly, there was slightly increased basal calcium flux (stimulated with media) of astrocytes pretreated with chronic METH (50 nM; blue bars) (Figure 3C) or infected with pseudotyped HIV-1 (500 RT; red bars) (Figure 3D) for 7 days. Restimulation of acute METH (250 μM) following chronic METH exposure or HIV-1 infection further elevated METH-induced intracellular calcium flux (∼3-fold vs. control, *p* < 0.01; *p* < 0.05 compared to respective media-stimulated controls). However, there were no statistically significant differences comparing chronic conditions to non-pretreated controls (white bars versus colored bars). Together, these data indicate that both prolonged METH exposure and HIV-1 infection may alter ER physiology by increasing both basal and METH-induced calcium flux, which could be contributing to the elevated levels of mitochondrial respiration following these conditions (Figure 2).

To further examine altered ER physiology and ER-associated signaling pathways in response to METH exposure and HIV-1 infection, protein expression of the three UPR arms (ATF6, IRE1α, and PERK), along with their classical ER stress negative regulating binding partner, BiP, was determined using Simple Wes (Figures 3E–H). To demonstrate variability across biological donors and conditions, representative Wes blots for two separate biological donors per condition are illustrated (Figures 3E,F). Notably, chronic METH (50 nM; blue bars) exposure significantly increased expression of UPR arm IRE1α by 50% (*p* < 0.05) without increasing BiP, ATF6, or PERK (Figure 3G), and HIV-1 infection (500 RT; red bars) induced significant increases in all arms and their binding partner (*p* < 0.05) (Figure 3H). Of note, most prominently elevated following HIV-1 infection was IRE1α with a 2-fold increase (*p* < 0.01). Contrary to chronic METH (50 nM) exposure, astrocytes stimulated with acute METH (5 μM; striped bars) for 8 h did not significantly increase IRE1α but instead increased expression of the other two arms, ATF6 and PERK (*p* < 0.01) (Supplementary Figure 2A). However, acute METH (5 μM) restimulation following chronic METH (50 nM) demonstrated altered responses in UPR arm protein induction (Supplementary Figures 2B–D). Indeed, acute METH-induced increases in ATF6 (Supplementary Figure 2B) and PERK (Supplementary Figure 2C) protein levels were significantly suppressed after chronic METH exposure (*p* < 0.05). Meanwhile, chronic METH-induced IRE1α (Supplementary Figure 2D) protein levels were also reduced back to control levels when restimulated with acute METH. These altered responses support a possible phenotypic shift in astrocyte UPR/ER stress responses following chronic METH exposure.

**FIGURE 4:**
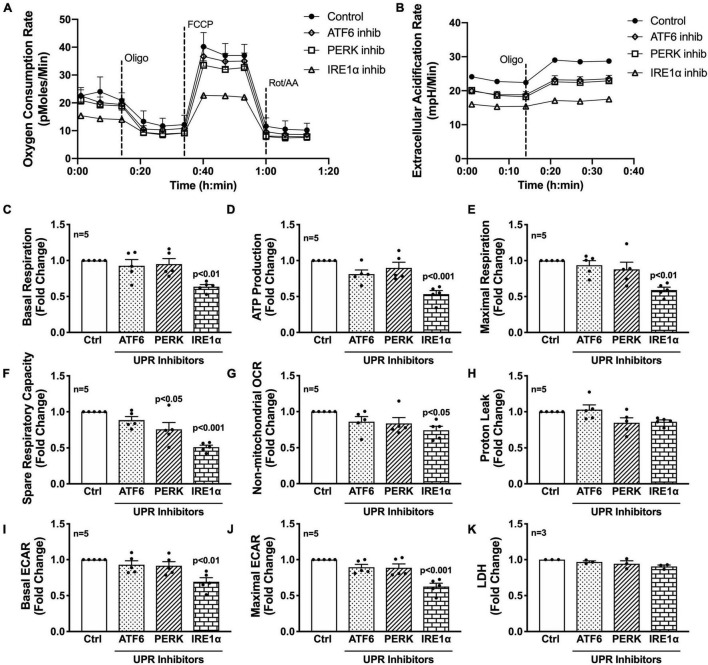
Inhibition of IRE1α decreases astrocyte metabolic function. **(A–K)** Astrocytes were treated with pharmacological inhibitors for the three UPR arms [ATF6 (AEBSF; 100 μM), PERK (GSK2606414; 1 μM), IRE1α (STF-083010; 60 μM)] for 3 h prior to **(A–J)** Seahorse Mito Stress Test for metabolic assessment or **(K)** extracellular lactate dehydrogenase (LDH) assay for cytotoxicity. Representative **(A)** OCR and **(B)** ECAR profile tracings from a single astrocyte donor were graphed over time. Compiled data from five separate biological donors quantifying fold changes in **(C)** basal respiration, **(D)** ATP production, **(E)** maximal respiration, **(F)** spare respiratory capacity, **(G)** non-mitochondrial OCR, **(H)** proton leak, **(I)** basal ECAR, and **(J)** maximal ECAR were graphed for statistical comparisons. Statistical significance was determined *via* one-way ANOVA followed by Tukey’s *post hoc* for multiple comparisons. Each dot on graphs represents the averaged data from a minimum of six replicates per biological donor.

Next, the three UPR arms were evaluated for their influence on astrocyte respiration to determine if they could be regulating astrocyte metabolic phenotypes. Astrocytes were treated with pharmacological inhibitors of the three UPR arms 3 h prior to assessment of metabolic function (Figure 4). Line tracings from representative experiments for OCR (Figure 4A) and ECAR (Figure 4B) readouts over time demonstrate decreased overall astrocyte metabolic function when any UPR arm was inhibited as compared to control rates. Subsequent parameter calculation from grouped OCR data in five separate astrocyte donors highlighted that IRE1α inhibition significantly impairs basal respiration (Figure 4C; *p* < 0.01), ATP production (Figure 4D; *p* < 0.001), maximal respiration (Figure 4E; *p* < 0.001), spare respiratory capacity (Figure 4F; *p* < 0.001), and non-mitochondrial OCR (Figure 4G; *p* < 0.05). Inhibition of PERK significantly inhibited spare respiratory capacity (Figure 4F; *p* < 0.05). Proton leak (Figure 4H) was not significantly altered from inhibiting any of the three UPR arms, suggesting that there were no direct effects of inhibitors on mitochondrial integrity. Instead, the effects on mitochondrial respiration were likely indirectly regulated though ER to mitochondrial signaling.

To gain further insight into the effects of the three arms on non-mitochondrial respiration, additional analysis using ECAR data allowed interpretation of basal and maximal glycolytic rates by calculating changes before and after injection of oligomycin (Figures 4I,J). Interestingly, both basal ECAR (Figure 4I) and maximal ECAR (Figure 4J) were impaired by IRE1α inhibition. Importantly, the effects of IRE1α inhibition on downregulating mitochondrial respiration and glycolysis were not due to changes in cytotoxicity as determined by LDH assay (Figure 4K). Moreover, two different pharmacological inhibitors for IRE1α were tested confirming specificity of IRE1α inhibition on astrocyte metabolic function (Supplementary Figure 3). Altogether, the data demonstrate IRE1α as a potential regulator of astrocyte metabolic function, both mitochondrial respiration and glycolysis. However, it remains to be determined if IRE1α may be regulating these two metabolic pathways through distinct mechanisms.

**FIGURE 5:**
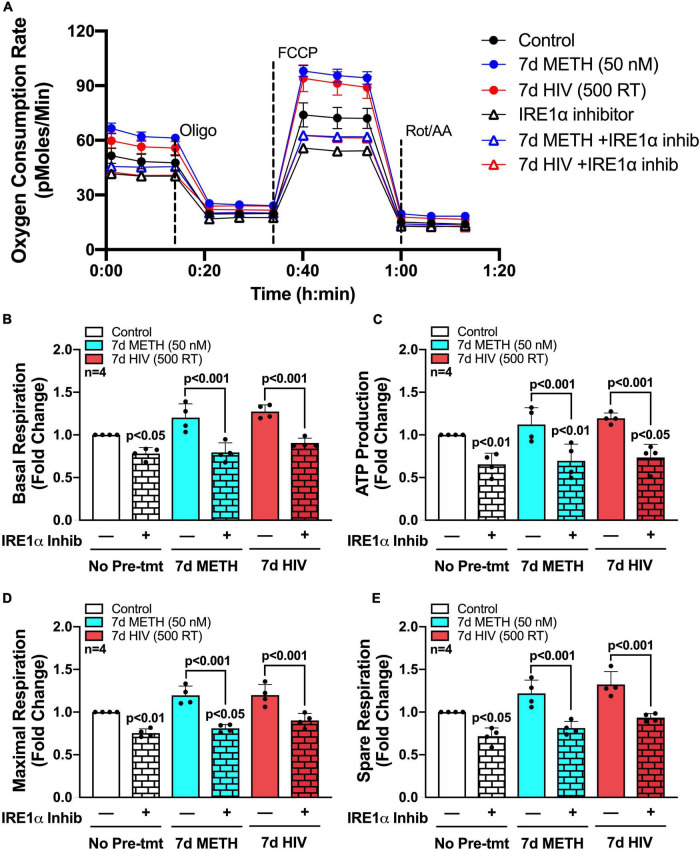
IRE1α inhibition can partially restore astrocyte metabolic function following HIV-1 infection and chronic METH exposure. **(A–E)** Astrocytes were treated with METH (50 nM; blue bars) or infected with pseudotyped HIV-1 (500 RT; red bars) for 7 days followed by IRE1α pharmacological inhibition (STF-083010, 60 μM) for 3 h prior to Seahorse Mito Stress Test. **(A)** Representative metabolic OCR profile tracing is illustrated from a single astrocyte donor. Compiled data quantifying fold changes in **(B)** basal respiration, **(C)** ATP production, **(D)** maximal respiration, and **(E)** spare respiratory capacity were graphed for statistical comparisons. Rescue experiments were performed twice in two separate biological donors to acquire four separate data sets. A minimum of six replicates were performed per experiment. Statistical significance was determined by one-way ANOVA followed by Tukey’s *post hoc* for multiple comparisons.

To determine if IRE1α inhibition could rescue the increased metabolic phenotypes that followed chronic METH exposure and HIV-1 infection, astrocytes were treated with chronic METH (blue bars) or infected with HIV-1 (red bars) prior to IRE1α inhibition (triangles and pattern bars) and subsequent metabolic analysis (Figure 5). Similar to above, OCR line tracings demonstrate that chronic treatments with METH exposure or HIV-1 infection increased astrocyte OCR, while IRE1α inhibition decreased respiratory activity (Figure 5A). Overall, inhibition of IRE1α significantly decreased (*p* < 0.001) METH- and HIV-1 induced increases in basal respiration (Figure 5B), ATP production (Figure 5C), maximal respiration (Figure 5D), and spare respiratory capacity (Figure 5E), indicating a potential restoration in respiratory phenotype. Indeed, HIV-1 infection with IRE1α inhibition was not significantly different from healthy controls (white bars) in basal, maximal, or spare respiration. Similarly, chronic METH with IRE1α inhibition was not significantly changed from healthy controls in both basal and spare respiration. However, IRE1α inhibition, independent of chronic treatment, significantly decreased ATP production (*p* < 0.05), suggesting an inability of IRE1α inhibition to completely rebalance astrocyte respiration following chronic METH exposure or HIV-1 infection.

**FIGURE 6:**
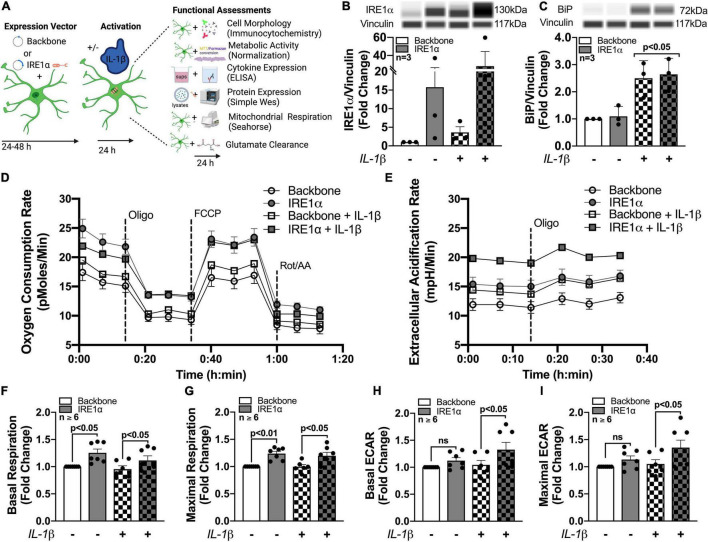
Astrocyte IRE1α regulates both mitochondrial respiration and glycolytic activity through distinct mechanisms. **(A–I)** Astrocytes were transfected with an IRE1α overexpression vector (gray bars) or backbone (white bars) and then treated with IL-1β for 24 h (checkered bars) prior to functional assessments. **(B,C)** Cellular lysates were collected and assayed by Simple Wes to quantify **(B)** IRE1α and **(C)** BiP expression levels. Vinculin was used as an internal control. **(D,F,G)** Mitochondrial respiration and **(E,H,I)** glycolytic activity were assessed by Seahorse metabolic assay. **(D)** OCR and **(E)** ECAR profile tracings from a representative astrocyte donor were graphed over time. Fold changes in **(F)** basal respiration, **(G)** maximal respiration, **(H)** basal ECAR, and **(I)** maximal ECAR were graphed for statistical comparisons. Individual dots on graphs represent the averaged data from a minimum of 6 replicates per biological donor. Significance was determined by one-way ANOVA and Tukey’s *post hoc* for multiple comparisons. Experimental Illustration was made with BioRender.com.

To isolate IRE1α regulatory function in astrocytes and model IRE1α upregulation following chronic METH exposure or HIV-1 infection, astrocytes were transfected with an IRE1α overexpression vector (gray bars) or backbone as a control (white bars) (Figures 6B,C). After recovery, astrocytes were treated with proinflammatory cytokine, IL-1β (20 ng/mL; checkered bars) for 24 h followed by various functional assessments (Figure 6A). In HAND, IL-1β is a prominent cytokine involved in astrocyte activation and has been extensively used to study the role of astrogliosis in HAND pathology (Roux-Lombard et al., 1989; Kou et al., 2009; Mamik et al., 2011; Nooka and Ghorpade, 2017; Edara et al., 2020b). Moreover, IL-1β is a potent inducer of ER stress in astrocytes (Nooka and Ghorpade, 2017). Thus, these studies can help illuminate the functional role of both IRE1α expression and activation in astrocytes. Increased protein expression of IRE1α following both overexpression transfection and IL-1β treatment was confirmed using Simple Wes (Figure 6B). Of the three separate biological donors tested, IL-1β treatment alone induced a ∼2 – 6-fold increase in IRE1α, while overexpression varied more substantially across biological donors with a ∼2 – 40-fold increase in expression. Moreover, overexpression with IL-1β treatment further increased expression in all donors with a ∼6 – 60-fold increase in IRE1α protein expression. As expected, BiP protein expression, which is classically used as an indicator for ER stress activation, was significantly increased following IL-1β treatment (*p* < 0.05) (Figure 6C). However, overexpression of IRE1α had no effect on BiP expression.

The effects of IRE1α overexpression on astrocyte metabolic phenotype in IL-1β-stimulated and unstimulated conditions were assessed by Cell Mito Stress Test (Figures 6D–I). Line tracings of OCR (Figure 6D) and ECAR (Figure 6E) readouts from representative astrocyte donors emphasize a unique discrepancy of the regulatory effects or IRE1α expression on astrocyte metabolic function in IL-1β-stimulated versus unstimulated conditions. Basal and maximal mitochondrial respiration, calculated from OCR, were significantly increased (∼25%; *p* < 0.05) in astrocytes when IRE1α was overexpressed compared to backbone controls, independent of IL-1β treatment (Figures 6F,G). However, basal and maximal glycolytic activities, calculated from ECAR, were only significantly increased (∼25%; *p* < 0.05) by IRE1α overexpression in astrocytes when stimulated by IL-1β (Figures 6H,I). Additional parameters calculated from OCR data, including spare respiratory capacity (Supplementary Figure 4A) ATP production (Supplementary Figure 4B), non-mitochondrial respiration (Supplementary Figure 4C), and proton leak (Supplementary Figure 4D), followed similar increased trends when IRE1α was overexpressed in astrocytes. However, only proton leak in IL-1β-unstimulated astrocytes was significantly elevated (∼25%; *p* < 0.05). Altogether, these findings confirm IRE1α as a regulator of astrocyte metabolic phenotype and support distinct mechanistic differences between IRE1α-mediated regulation on mitochondrial respiration versus glycolytic activity.

**FIGURE 7:**
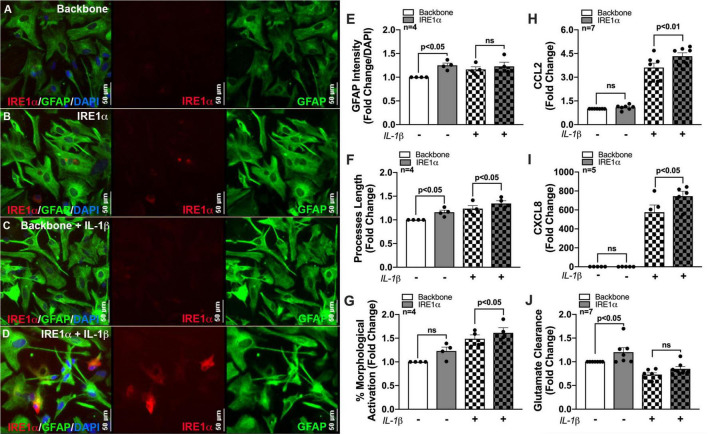
IRE1α overexpression augments cytokine expression and increases glutamate clearance in human astrocytes. Backbone and IRE1α transfected astrocytes were treated with IL-1β for 24 h. **(A–D)** Cells were immunolabeled with antibodies specific for IRE1α (red) and the astrocyte marker glial fibrillary acidic protein (GFAP, green). Nuclear DNA was labeled with DAPI (blue). **(E–G)** Experiments in four separate biological donors were analyzed to quantify morphological activation. Individual dots on graphs represent compiled fold-changes calculated from duplicate wells and/or triplicate images per condition for each biological astrocyte donor. **(E)** GFAP intensity was measured across full-well scans using SoftMax Pro and normalized to DAPI. **(F)** Process length of individual astrocytes was manually traced and measured using ImageJ Software. **(G)** Percent morphological activation was calculated based on the number of astrocytes presenting with ‘reactive’ morphology divided by the total number of astrocytes imaged. **(H)** CCL2 and **(I)** CXCL8 levels were assessed by an ELISA, and expression was normalized to metabolic activity prior to calculating fold changes to backbone. **(J)** Astrocytes were treated with 400 nM glutamate for 24 h. Remaining glutamate levels were quantified by fluorescent assay to calculate% glutamate clearance followed by fold change for each individual donor. Individual dots on graphs represent compiled data from triplicate experiments per biological astrocyte donor. Significance was determined by one-way ANOVA and Tukey’s *post hoc* for multiple comparisons.

Previous studies linking IRE1α to metabolic regulation correlate these changes to cellular activation/inflammation suggesting IRE1α-mediated induction of mitochondrial respiration is essential for immune activation (Dong et al., 2019; Lara-Reyna et al., 2019; Abuaita et al., 2021). In fact, IRE1α has commonly been highlighted as a regulator of inflammation and immune responses to infections (Abuaita et al., 2015, 2018, 2021; Dong et al., 2019; Lara-Reyna et al., 2019; Wheeler et al., 2019). However, this functional linkage between metabolism and inflammation has yet to be established using an IRE1α overexpression model or in astrocytes. As astrocyte-mediated neuroinflammation is a hallmark of HAND pathology, we wanted to evaluate the potential of IRE1α in regulating astrocyte inflammatory phenotype. Thus, using our established overexpression model, we merged our lab’s decade-long optimized protocol for characterizing the activation of human astrocytes (Edara et al., 2020a; You et al., 2020). Immunocytochemistry staining revealed an activated astrocyte morphology post treatment with IL-1β, as indicated by an increased prominence in processes and cell body constriction and increased appearance in GFAP intensity. Moreover, comparing astrocytes overexpressing IRE1α to respective untreated and IL-1β treated backbone groups demonstrated IRE1α may be contributing to astrogliosis morphology (Figures 7A–D). Quantification of astrocyte reactivity demonstrated IL-1β stimulation did not significantly affect GFAP expression (Figure 7E), but significantly increased astrocyte process length (Figure 7F; *p* < 0.001; statistics not shown on graph) and overall percent of astrocytes demonstrating morphological activation (Figure 7G; *p* < 0.01; statistics not shown on graph). Furthermore, IRE1α overexpression in the absence of IL-1β stimulation significantly increased GFAP intensity and astrocyte process length (*p* < 0.05) as compared to backbone controls. Similarly, IRE1α overexpression with IL-1β stimulation also increased process length and morphological activation (*p* < 0.05) as compared to activated backbone controls. Delving into astrocyte inflammatory functions, our lab has well-established that astrocyte CCL2 (Figure 7H) and CXCL8 (Figure 7I) cytokine release is strongly upregulated in response to IL-1β (*p* < 0.01; statistics not shown on graph); however, these responses were significantly augmented when IRE1α was overexpressed (*p* < 0.05). Finally, a key functional impairment of astrocytes in neuropathology is a decrease in glutamate clearance efficacy, which can contribute to excitotoxicity in neurons. When activated by IL-1β, astrocytes were unable to clear glutamate as efficiently in comparison to untreated cultures (*p* < 0.01; statistics not shown on graph) (Figure 7J). Interestingly, overexpression of IRE1α significantly enhanced astrocyte glutamate uptake in IL-1β unstimulated cultures (*p* < 0.05). While IL-1β-stimulated astrocytes had a slight increase when IRE1α was overexpressed, these results were not statistically significant.

## Discussion

**SCHEME 1:**
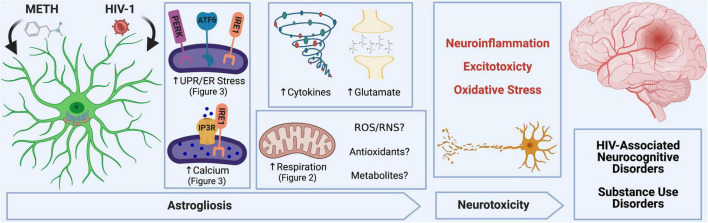
METH and HIV-1 alter astrocyte function to induce neurotoxicity, which determines the pathology of HAND and METH use disorders. Neuroinflammation, oxidative stress and glutamate excitotoxicity are hallmarks of neurodegenerative pathology and are all propagated by astrocyte dysfunction. During a pathological challenge, such as METH exposure and HIV-1 infection, astrocytes became reactive leading to a shift in their neurotrophic functions to become neurotoxic. This reactive state, often termed as astrogliosis, is characterized by an increased inflammatory phenotype which promotes neuroinflammation. Moreover, decreased provision of essential metabolic and antioxidant support to neurons and increased release of toxic radicals such as reactive oxygen and nitrogen species (ROS/RNS) are triggers for oxidative stress. Finally, excitotoxicity arises from an impaired uptake of excess glutamate between synaptic junctions. Our studies emphasize the role of astrocyte ER-associated mechanisms and mitochondrial dysfunction following METH exposure and HIV-1 infection as potential underlying mechanics controlling astrocyte dysfunction and astrocyte-associated neurodegeneration. Experimental Illustration was made with BioRender.com.

In the context of METH exposure and HIV-1-relevant stimuli, astrocytes become activated, which can lead to neurotoxic consequences and contribute to METH use and HAND pathology (Scheme 1). Major mechanisms for which astrocytes can inflict neuronal damage following METH and/or HIV-1-relevant stimuli include astrocyte-associated neuroinflammation and glutamate excitotoxicity, which our team and others have also previously characterized (Cisneros and Ghorpade, 2012, 2014; Borgmann and Ghorpade, 2015; Cisneros et al., 2020; Edara et al., 2020a). Moreover, astrocyte mitochondrial dysfunction may threaten the ability of astrocytes to provide essential metabolic and antioxidant support to neurons and could also contribute to the release of toxic reactive oxygen and nitrogen species (ROS/RNS) to propagate oxidative stress.

Identifying a cellular or molecular regulator of astrocyte dysfunction will be essential to ensure neuronal fitness and restore CNS homeostasis when facing a neuropathological challenge, such as METH use and HAND. The current study was centered on evaluating the effects of *chronic low-dose* METH exposure and HIV-1 *infection* on astrocyte ER and mitochondrial homeostasis and then examining ER-associated mechanisms as potential therapeutic targets for astrocyte dysfunction. These findings illustrate that astrocytes have increased basal and maximal respiration, spare respiratory capacity and non-mitochondrial OCR following HIV-1 infection or chronic low-dose METH exposure. Increased metabolic activity suggests that astrocytes may need to compensate during HIV/METH-associated stress to meet the increased energic and antioxidant demands. However, increased metabolic respiration did not translate to increased energy output (ATP production). Coupled with an increase in proton leak, these results suggest that astrocyte mitochondrial function was impaired by chronic METH exposure or HIV-1 infection.

Importantly, increased mitochondrial respiratory activity was associated with increased calcium flux and UPR protein expression following HIV-1 infection and METH exposure in primary human astrocytes. Briefly, HIV-1 infection increased expression of all three UPR arms and a key protein-folding chaperone, highlighting profound implications on the long-term restructuring of ER and MAMs during HIV-1/HAND pathology. Acute METH exposure also induced expression of all three UPR messengers, but chronic METH only significantly increased protein expression of IRE1α. Moreover, chronic METH exposure dysregulated both calcium flux and UPR induction when astrocytes were rechallenged with acute METH, supporting altered ER and MAM function and physiology as key regulators in astrocyte dysfunction.

Unique in both HIV-1 and METH paradigms, IRE1α was prominently upregulated. Distinct from the other UPR arms, IRE1α has previously been shown to regulate mitochondrial respiratory activity and ROS production as a mechanism to control cellular immune and inflammatory responses in a variety of different cell types (Abuaita et al., 2015, 2018, 2021; Dong et al., 2019). Moreover, some studies have also highlighted a novel role of IRE1α in the ER-mitochondrial interface as a key regulator of ER-mitochondrial calcium transfer as the potential mechanism for IRE1α regulation of mitochondrial function. Interestingly, these reports differ in the identified interaction in which IRE1α regulates ER-mitochondrial calcium transfer. For example, IRE1α has been shown to both directly (Carreras-Sureda et al., 2019) and indirectly (Son et al., 2014, 2021) regulate IP_3_R stability in the ER membrane as well as with σ-1R (Mori et al., 2013) in the ER lumen. Indeed, σ-1R is a known astrocyte binding receptor of METH and is implicated as a regulator of METH-mediated astrocyte dysfunction (Zhang et al., 2015). Could these regulatory mechanisms be in association with IRE1α? Notably, recent studies have illuminated IRE1α/σ-1R signaling as potential therapeutic targets for dysfunctional immune and inflammatory pathologies (Rosen et al., 2019).

Our findings suggest IRE1α activity can regulate multiple aspects of astrocyte function including ER stress, mitochondrial respiration, glycolysis, inflammation, and glutamate clearance. A summary of IRE1α-associated regulation of astrocyte function is illustrated in Table 1. The canonical model for UPR/ER stress activation occurs in response to accumulated unfolded or misfolded proteins, where each UPR arm can orchestrate unique cellular mechanisms to reprogram transcriptional profiles and restore proteostasis. These same messengers can also function to initiate apoptosis if stress is unable to be resolved (Ghemrawi and Khair, 2020). However, as astrocytes are resilient to HIV-1/METH-induced stress, *chronic* upregulation of IRE1α may instead present as key regulator of astrogliosis beyond its classical UPR/ER stress function. The molecular mechanisms and functional diversity of IRE1α have been previously reviewed (Bashir et al., 2021). Briefly, IRE1α has both kinase and endoribonuclease enzyme activity. There are two divergent classical pathways associated with IRE1α’s UPR functions: X-box binding protein 1 (XBP1) and regulated IRE1-dependent decay (RIDD). Notably the IRE1α/XBP1 pathway is primarily associated with upregulation of UPR gene transcription but is also implicated in metabolic disorders and cancer pathology. The IRE1α/RIDD signaling branch primarily regulates IRE1α-mediated mRNA degradation but is also implicated in inflammatory and apoptotic signaling. Importantly, IRE1α can also transmit information independent of its enzymatic activity. A new concept defined as a “UPRsome” has now been introduced, highlighting IRE1α as a potential signaling scaffold for a series of protein interactions to regulate cellular fate (Riaz et al., 2020; Urra et al., 2020; Bashir et al., 2021).

**TABLE 1:** Astrocyte IRE1α regulates ER stress, mitochondrial respiration, glycolysis, inflammation, and glutamate clearance.

IRE1α	Activity		Over-expression	Over- expression + IL-1β
			
	Inhibition	IL-1β		
ER Stress (BiP)	N/A	↑	–	↑↑
Mitochondrial Function (OCR)	↓	–	↑	↑
Glycolytic Activity (ECAR)	↓	–	–	↑↑
Inflammation (CCL2 and CXCL8)	N/A	↑	–	↑↑
Glutamate Clearance	N/A	↓	↑	–

In our studies, inhibition of IRE1α impaired astrocyte metabolic function, both mitochondrial respiration and glycolysis. Activation of IRE1α *via* IL-1β stimulation (without IRE1α overexpression) increased astrocyte ER stress and inflammation while perturbing astrocyte glutamate uptake yet did not appear to significantly influence astrocyte metabolic function. Interestingly, overexpression alone led to increased mitochondrial OCR and increased glutamate uptake. As astrocytes convert glutamate into glutamine, which can be then used as a source for metabolic activities (Schousboe et al., 2014), it is possible IRE1α-mediated increased glutamate uptake may be related to the increased OCR. As stated above, IRE1α/XBP1 signaling has been implicated in metabolic homeostasis. In accordance, it has previously been shown that IRE1α/XBP1 signaling can regulate glutamate receptor trafficking in neurons of *C. elegans* (Shim et al., 2004). Of note, these observations specifically proposed that partial activation of IRE1α/XBP1 signaling for glutamate receptor trafficking occur in the absence of classical ER stress. Also of note, the effects of IRE1α overexpression on astrocyte glutamate uptake were contrary to expected outcomes; as glutamate clearance is classically impaired when astrocytes have an increased inflammatory phenotype (such as IL-1β stimulation). These findings suggest that different IRE1α-mediated mechanisms or pathways may be at play in regulating astrocyte inflammatory versus tripartite synaptic functions. Finally, combination of IRE1α overexpression with IL-1β stimulation amplified all functional outcomes except glutamate uptake, which instead may have had neutralizing effects.

The function of astrocyte IRE1α on mitochondrial and glycolytic activities may also follow similar signaling discrepancies. For example, IRE1α inhibition regulated both mitochondrial and glycolytic activity; however, overexpression alone only regulated mitochondrial respiration. While IRE1α overexpression with IL-1β stimulation increased both mitochondrial and glycolytic activity, the effects of overexpression and IL-1β activation were compounded for glycolysis, but not mitochondrial respiration. These findings suggest that astrocyte glycolysis and astrocyte mitochondrial respiration may be differentially regulated by IRE1α. Thus, while our studies highlight astrocyte IRE1α as a key regulator of ER stress, mitochondrial respiration, glycolysis, inflammation, and glutamate clearance, distinct signaling pathways and/or mechanisms may be involved in regulating these IRE1α-mediated functional outcomes. For example, some outcomes may be regulated by IRE1α classical UPR/ER stress signaling cascades; some may be through direct protein interactions in the UPRsome, while others may be regulated by IRE1α non-conical mechanisms within the ER-mitochondrial interface. Further studies are needed to delineate the mechanisms of how IRE1α regulates these astrocyte phenotypes.

Regardless, the biological relevance and potential therapeutic targeting of astrocyte IRE1α to combat HAND, METH use disorders and other neurodegenerative pathologies remain unclear. Targeting IRE1α must be balanced to diminish neurotoxic outcomes, while also preserving its neurosupportive functions is critical to restore astrocyte cellular homeostasis. Since decreased glutamate clearance is critical mechanism of astrocyte-associated neurotoxicity, targeting IRE1α expression may be a novel therapeutic strategy. However, beneficial functional changes in one astrocyte function, could be detrimental to other critical functions. For instance, it is unknown if increased mitochondrial respiration would be associated with neuroprotective (i.e., production and release of metabolites, antioxidants) or neurotoxic consequences (i.e., release of toxic radicals and insufficient neuronal support). If IRE1α-mediated increases in astrocyte metabolic function can enhance essential metabolite and antioxidant provision to neurons without provoking oxidative stress, targeting astrocyte IRE1α may help optimize the coupling between astrocytes and neurons to promote neuronal fitness during a neuropathic challenge. Another caveat, however, are the effects of IRE1α on inflammation. Indeed, inflammation is an immunological response intended to protect a host against injury or infection. However, chronic inflammation can often be the unintended culprit causing pathological tissue damage in many different diseases, like that in HAND.

In summary, the prospect of targeting ER stress and the three UPR arms to combat neurodegeneration has been widely explored as previously reviewed (Ghemrawi and Khair, 2020). It is now known that the three arms have unique non-canonical functions within the MAM interface. Moreover, the IRE1α arm has additional stress-associated and non-canonical complexity within the UPRsome. Our group recently published a review highlighting the potential of targeting MAMs in neurodegeneration with specific attention to ER stress, calcium dysregulation, and mitochondrial dysfunction in the context of HAND (Proulx et al., 2021). These MAM-mediated mechanisms are prominent perpetrators underlying neuropathology among others including autophagy, inflammation, and apoptosis. Here, we highlight the importance of astrocyte IRE1α as a key regulator of ER stress, mitochondrial respiration, glycolysis, inflammatory responses, and glutamate clearance during chronic HIV infection, METH exposure and neuroinflammation. Moreover, these findings suggest that both canonical and non-canonical UPR mechanisms of astrocyte IRE1α. For IRE1α to be a targetable entity in astrocytes, additional studies will need to delineate the mechanistic properties of how IRE1α regulates these different aspects of astrocyte function. Based on our findings, among other, targeting IRE1α will likely require manipulations that target specific interactions, activities, and/or pathways that best promote neuroprotective properties while preventing neurotoxic properties. Alternatively, exploration into upstream or downstream factors could provide additional therapeutic application.

## Data Availability Statement

The raw data supporting the conclusions of this article will be made available by the authors, without undue reservation.

## Author Contributions

JP performed the experiments with excellent technical assistance from SS and guidance and supervision from KB. JP drafted the manuscript text and figures with guidance and supervision from KB. I-WP provided additional input with data interpretation and scientific conceptualization. All authors participated in editing or revising the article and agreed to be accountable for the content of the work.

## Conflict of Interest

The authors declare that the research was conducted in the absence of any commercial or financial relationships that could be construed as a potential conflict of interest.

## Publisher’s Note

All claims expressed in this article are solely those of the authors and do not necessarily represent those of their affiliated organizations, or those of the publisher, the editors and the reviewers. Any product that may be evaluated in this article, or claim that may be made by its manufacturer, is not guaranteed or endorsed by the publisher.

## Abbreviations

ART: antiretroviral therapy
ATF6: activating transcription factor 6
AUC: area under the curve
BBB: blood brain barrier
BCA: bicinchoninic acid
BiP: binding immunoglobulin protein
CCL2: C-C motif chemokine ligand 2
CNS: central nervous system
DAPI: 4′,6-diamidino-2-phenylindole
CXCL8: C-X -C motif chemokine ligand 8
ECAR: extracellular acidification rate
ELISA: enzyme-linked immunosorbent assays
ER: endoplasmic reticulum
ETC: electron transport chain
FCCP: carbonyl cyanide-4 (trifluoromethoxy) phenylhydrazone
GCaMP6s: GFP-calmodulin calcium sensor
GFAP: glial fibrillary acid protein
gp120: glycoprotein 120
grp75: glucose-regulated protein 75 kDa
pHEF: p-human elongation-factor
HEK: human embryonic kidney
HIV-1: human immunodeficiency virus type 1
HAND: HIV-associated neurocognitive disorders
IL-1 β: interleukin 1 β
IP_3_R: inositol 1,4,5-triphosphate receptors
IRE1 α: inositol-requiring enzyme 1 α
LDH: lactate dehydrogenase
MAM: mitochondria-associated ER membrane
MCU: mitochondrial calcium uniporter
METH: methamphetamine
MFN: mitofusin
MPER: mammalian protein extraction buffer
MTT: 3-(4,5-dimethylthiazol-2-yl)-2,5-diphenyltetrazolium bromide
Nef: negative factor
OCR: oxygen consumption rate
Oligo: oligomycin
PERK: protein kinase RNA-like endoplasmic reticulum kinase
PHA: primary human astrocytes
PLWH: people living with HIV
RIDD: regulated IRE1-dependent decay
ROS/RNS: reactive oxygen and nitrogen species
Rot/AA: rotenone/antimycin A
RT: reverse transcriptase
σ -1R: sigma-1 receptor
Tat: transactivator of transcription
UPR: unfolded protein response
VAPB: vesicle-associated membrane protein-associated protein B
VDAC: voltage-dependent anion-selective channel
VSVg: vesicular stomatitis virus glycoprotein
XBP1: X-box binding protein 1.
