# Supplementary material for: A Non-Canonical Role for IRE1α Links ER and Mitochondria as Key Regulators of Astrocyte Dysfunction: Implications in Methamphetamine use and HIV-Associated Neurocognitive Disorders
Source: Front Neurosci. 2022 Jun 17;16:906651. doi: 10.3389/fnins.2022.906651 (PMC9247407; doi:10.3389/fnins.2022.906651)
Supplement: Supplementary file 1 [file Image_1.pdf]

Supplementary Figures

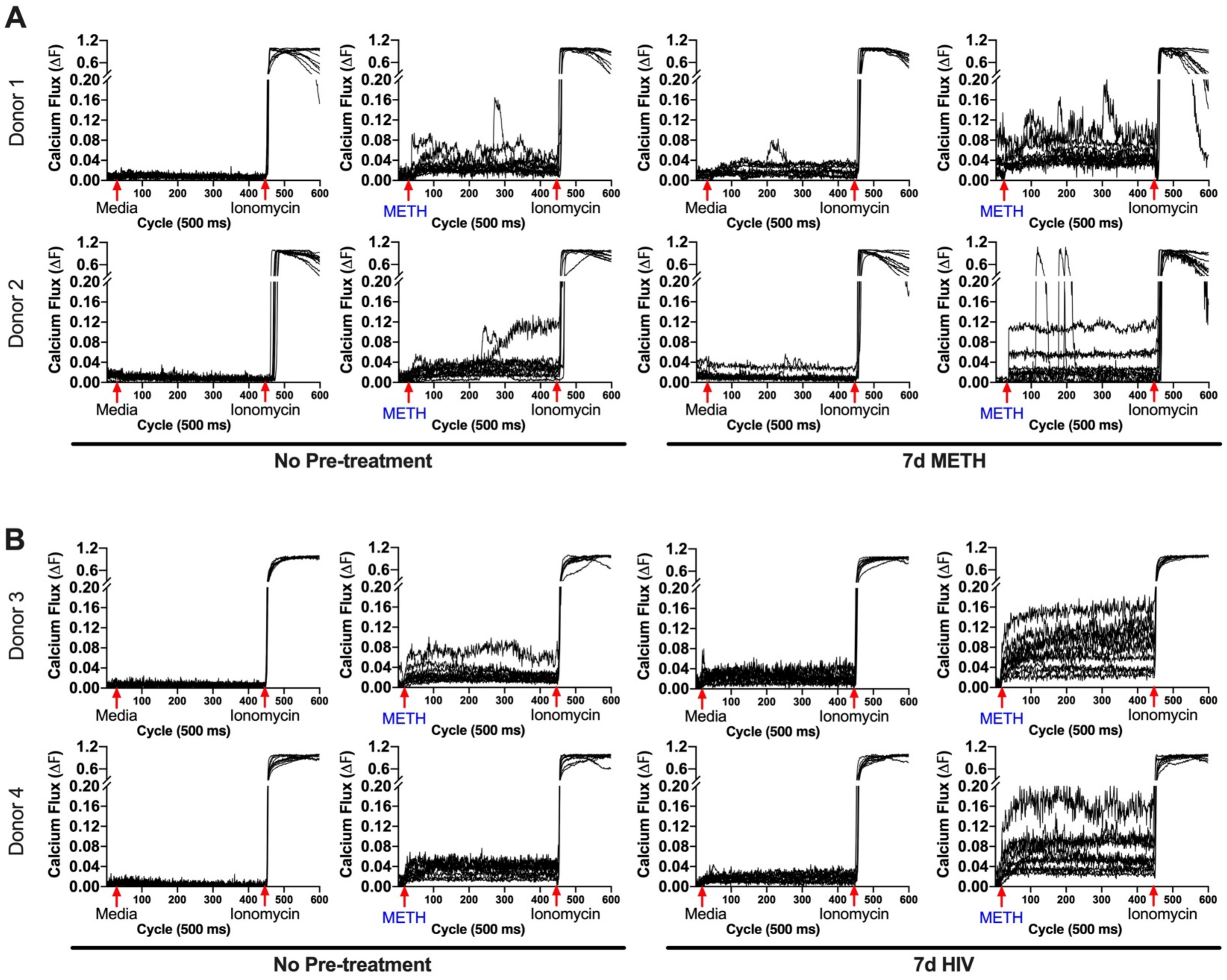

Supplementary Figure 1: Chronic METH or HIV-1 infection increase basal and METH-induced astrocyte calcium flux. Astrocytes were untreated (No Pre-treatment) or treated with **(A)** METH (50 nM) or **(B)** infected with pseudotyped HIV-1 (500 RT) for 7 d and transfected with a GFP/calmodulin calcium sensor (GCaMP6s) for 48 h prior to calcium flux analysis. Time series confocal imaging was used to measure changes in fluorescence every 500 ms for a total of 5 min (600 cycles). Astrocyte were stimulated with control media or METH (250 μM) at 20 cycles (10 sec) and ionomycin (10 μM) at 450 cycles (225 sec) as positive control for maximum fluorescence. Changes in astrocyte calcium flux (ΔF) was calculated by: $\Delta F=(F-F_0)/(F_{max}-F_0)$, where F is the fluorescence intensity at any given time; F_0 is the baseline (1 – 20 cycles) fluorescence intensity, and F_{max} is the maximum fluorescence intensity when exposed to ionomycin (450 – 600 cycles). **(A-B)** Calcium flux line tracings illustrate the ΔF at any given time point. A total of 10 cells per condition are graphed from two separate donors per chronic treatment paradigm.

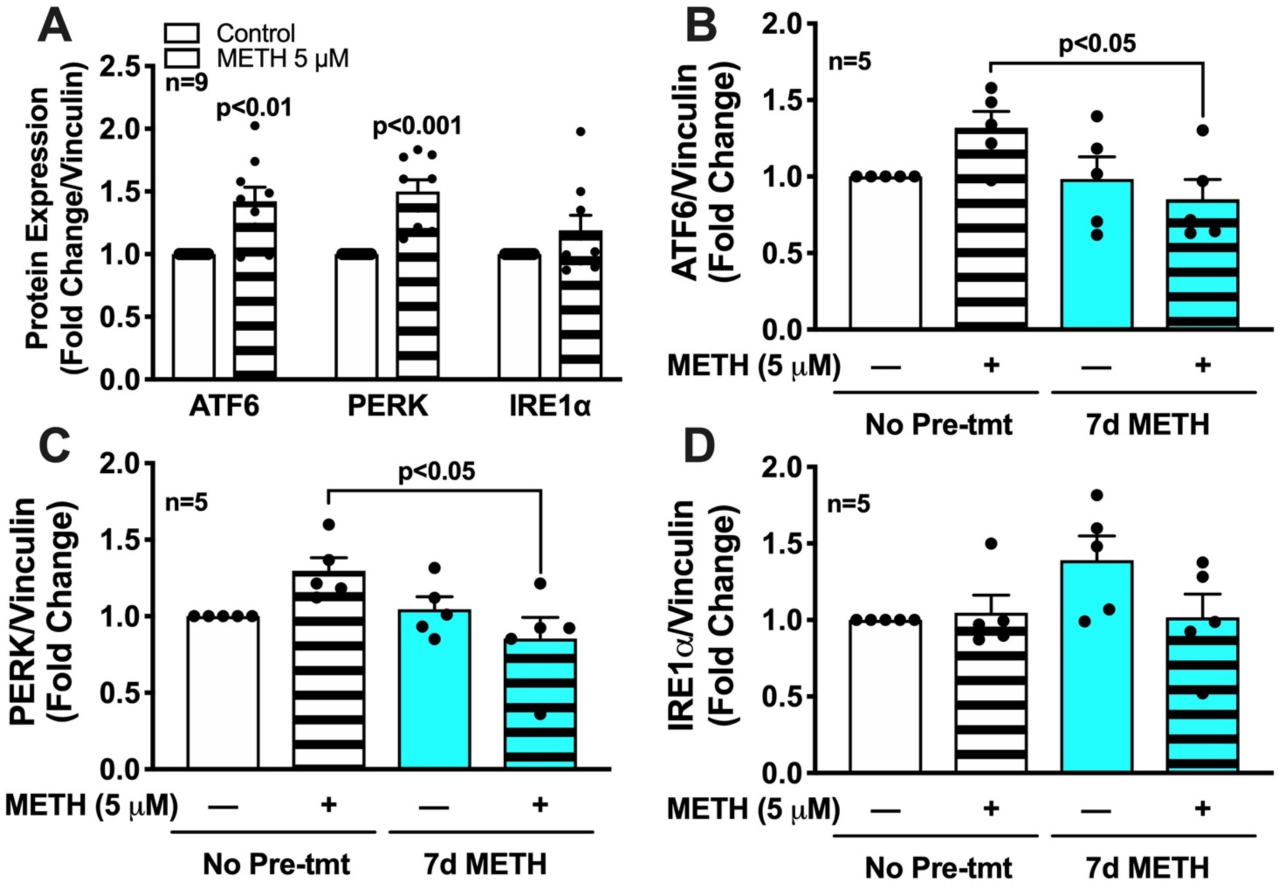

Supplementary Figure 2: Astrocyte UPR expression is altered by acute and chronic METH exposure. Astrocytes were treated with acute METH (5 uM) for 8 h (striped bars) and/or chronic METH (50 nM) for 7 d (blue bars) prior to protein lysate collection. Protein expression of **(A, B)** ATF6, **(A, C)** PERK, **(A, D)** IRE1α was measured *via* Simple Wes. Each dot represents separate biological donors. Statistics was performed using **(A)** ratio-paired t-tests for individual targets per condition or **(B-D)** one-way ANOVA followed by Tukey's post-hoc for multiple comparisons.

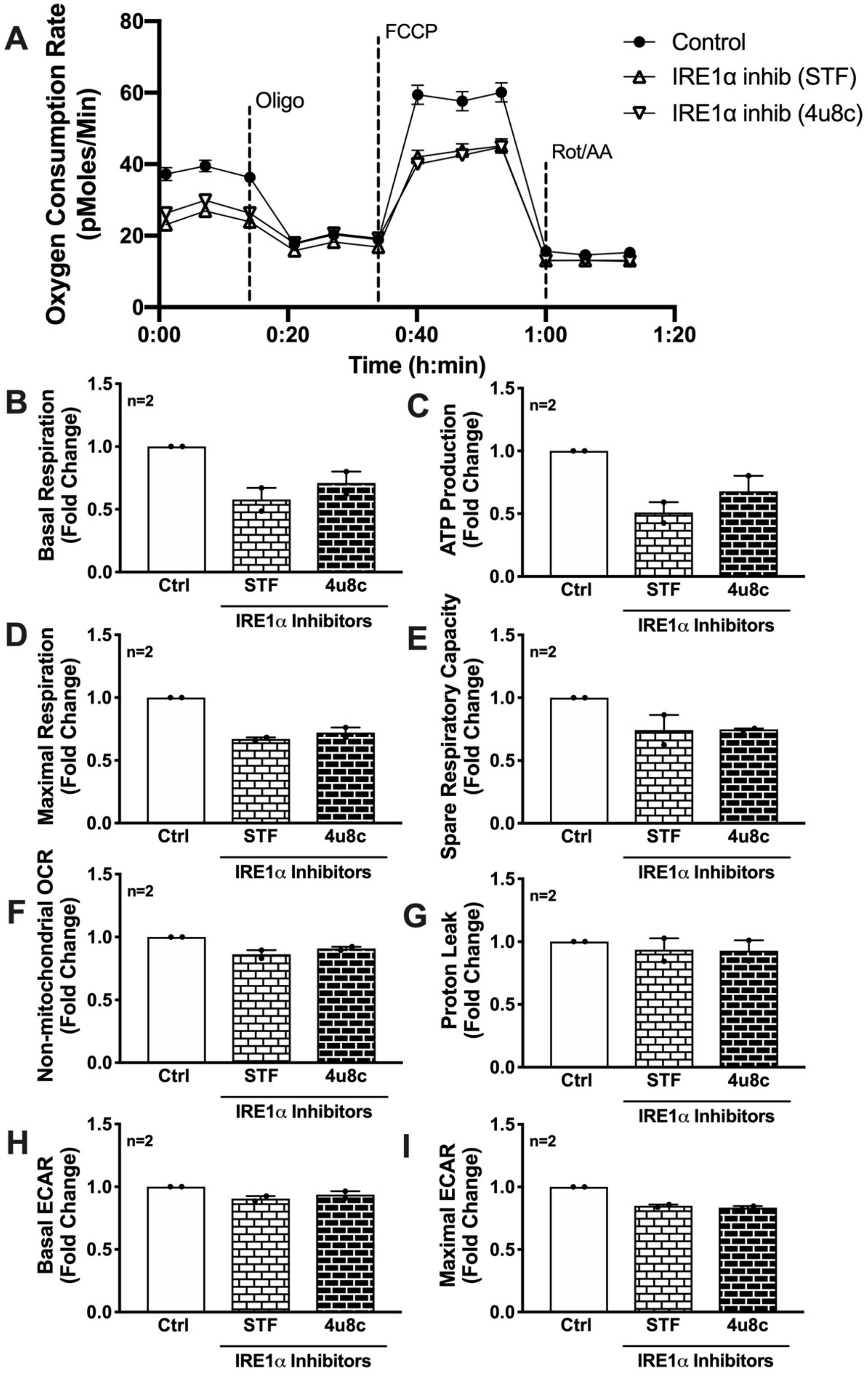

Supplementary Figure 3: Inhibition of IRE1α decreases astrocyte metabolic activity. (A-I) Astrocytes were treated with pharmacological inhibitors for IRE1α (STF-083010, 60 μM; 4μ8c, 50 μM) for 3 h prior to Seahorse Mito Stress Test. **(A)** Representative metabolic OCR profile tracing from a single astrocyte donor. Compiled data from two separate biological donors quantifying fold changes in **(B)** basal respiration, **(C)** ATP production, **(D)** maximal respiration, **(E)** spare respiratory capacity, **(F)** non-mitochondrial OCR **(G)** proton leak **(H)** basal ECAR, and **(I)** maximal ECAR were graphed.

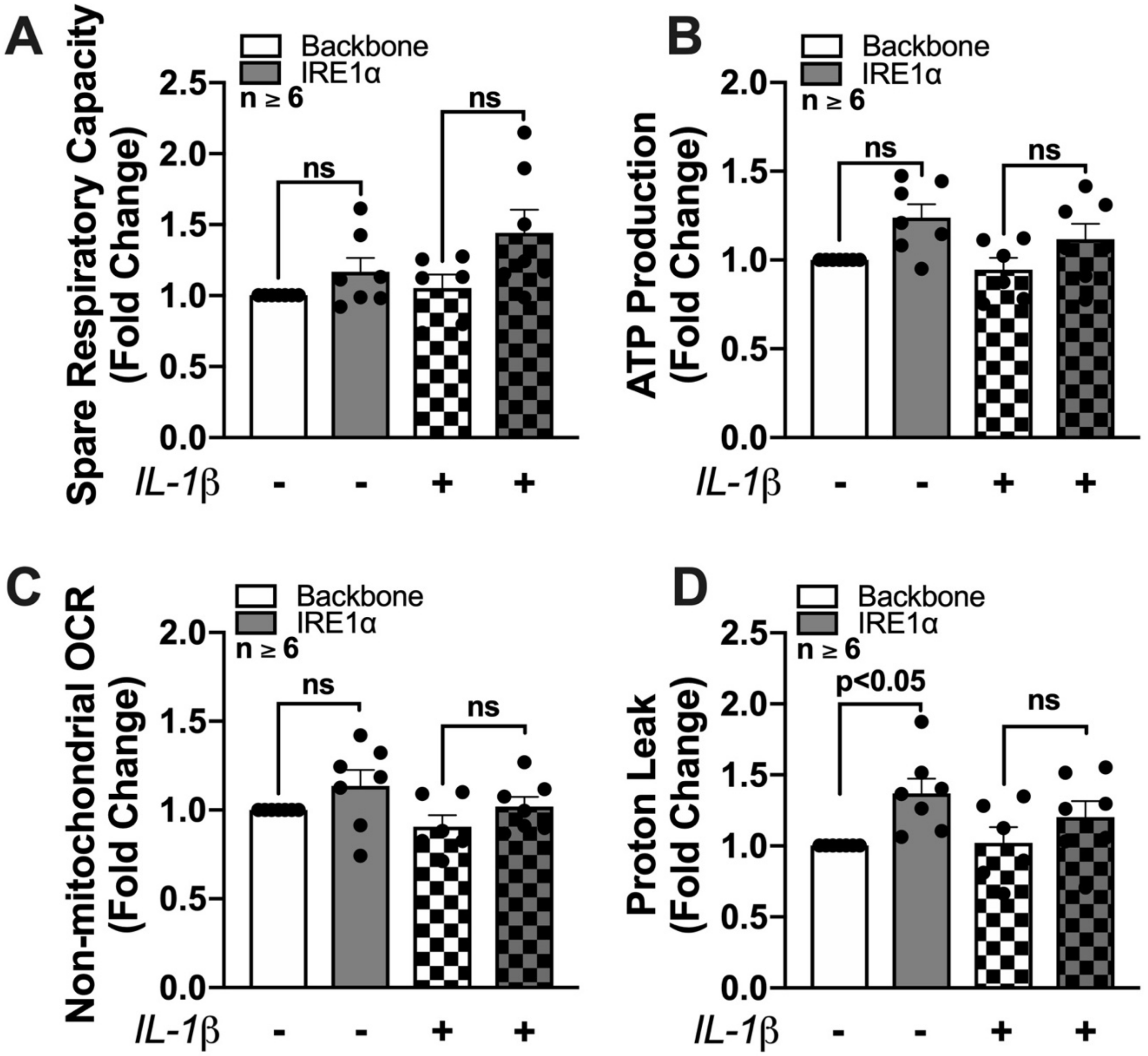

Supplementary Figure 4: IRE1α overexpression increases astrocyte metabolic activity. (A-D) Astrocytes were transfected with an IRE1α overexpression vector (gray bars) or backbone (white bars) and then treated with IL-1β for 24 h (checkered bars) prior to Seahorse Mito Stress Test assay. Compiled data from at least five separate biological donors quantifying fold changes in **(A)** spare respiratory capacity, **(B)** ATP production, **(C)** non-mitochondrial OCR, and **(D)** proton leak were graphed for statistical comparisons. Each dot on graphs represents a separate biological astrocyte donor. Significance was determined by one-way ANOVA and Tukey's post-hoc for multiple comparisons.
